# Exploring the therapeutic mechanism of Yuebi decoction on nephrotic syndrome based on network pharmacology and experimental study

**DOI:** 10.18632/aging.206116

**Published:** 2024-09-20

**Authors:** Tianwen Yao, Qingliang Wang, Shisheng Han, Yanqiu Xu, Min Chen, Yi Wang

**Affiliations:** 1 Department of Nephrology, Yueyang Hospital of Integrated Traditional Chinese and Western Medicine, Shanghai University of Traditional Chinese Medicine, Shanghai 200437, China; 2 Shanghai Jing'an District Hospital of Traditional Chinese Medicine, Shanghai 200072, China

**Keywords:** Yuebi decoction, nephrotic syndrome, podocyte injury, network pharmacology, molecular docking

## Abstract

Objective: This study aimed to explore the material basis of YBD and its possible mechanisms against NS through network pharmacology, molecular docking, and *in vivo* experiment.

Methods: Active ingredients and potential targets of YBD were obtained through TCMSP and SwissTargetPrediction. NS-related targets were obtained from GeneCards, PharmGKB, and OMIM databases. The herb-ingredient-target network and PPI network were constructed by Cytoscape 3.9.1 and STRING database. GO and KEGG analyses were performed by DAVID database and ClueGO plugin. The connection between main active ingredients and core targets were revealed by molecular docking. To ascertain the effects and molecular mechanisms of YBD, a rat model was established by PAN.

Results: We collected 124 active ingredients, 269 drug targets, and 2089 disease targets. 119 overlapping were screened for subsequent analysis. PPI showed that AKT1, STAT3, TRPC6, CASP3, JUN, PPP3CA, IL6, PTGS2, VEGFA, and NFATC3 were potential therapeutic targets of YBD against NS. Through GO and KEGG analyses, it showed the therapeutic effect of YBD on NS was closely involved in the regulation of pathways related to podocyte injury, including AGE-RAGE signaling pathway in diabetic complications and MAPK signaling pathway. Five key bioactive ingredients of YBD had the good affinity with the core targets. the experiment confirmed the renoprotective effects of YBD through reducing podocyte injury. Furthermore, YBD could downregulate expressions of PPP3CA, STAT3, NFATC3, TRPC6, and AKT1 in rats.

Conclusions: YBD might be a potential drug in the treatment of NS, and the underlying mechanism is closely associated with the inhibition of podocyte injury.

## INTRODUCTION

Nephrotic syndrome (NS) is defined by severe proteinuria, hypoalbuminemia, hyperlipemia and edema, which is often associated with acute kidney injury (AKI), thromboembolism and infection [1]. As one of the most common glomerular diseases [2], NS is characterized by the high morbidity, disability rate and death rate despite huge advances in its treatment [3]. For example, as a common pathological type of NS, minimal change disease (MCD) occupies a proportion of 15% in adults, and up to 70–90% in children [4]. Currently, the mechanism of NS is complex and controversial, but podocyte injury is regarded as the central event [5]. As the glomerular epithelial cell, podocyte has been considered as the final gatekeeper of glomerular filtration barrier [6]. Nowadays, glucocorticoids, calcineurin inhibitor, and rituximab are widely used in clinic, which may in turn cause a series of problems, such as serious adverse effects, economic pressure and frequent recurrence [7]. Therefore, exploring more advanced drugs is crucial in treating NS.

Recently, accumulated evidences further demonstrate traditional Chinese medicine (TCM) is applied in treating NS with therapeutic efficacy, low cost, and few side effects [8–10]. Yuebi Decoction (YBD), a famous traditional Chinese medicine compound, is composed of five Chinese medicinal materials, including *Ephedra sinica Stapf* (Mahuang, MH), *Gypsum Fibrosum* (Shigao, SG), *Zingiber officinale Rosc* (Shengjiang, SJ), *Glycyrrhiza uralensis Fisch* (Gancao, GC) and *Ziziphus jujuba Mill* (Dazao, DZ). Recently, YBD has been reported to show positive effects on decreasing proteinuria, increasing serum albumin, ameliorating renal function, reducing oxidative stress and inflammatory reaction [11, 12]. In clinical research reports, YBD has been proved to treat NS, and the total effective rate was up to 95.00% [13]. Therefore, YBD is widely used in the treatment of NS. MH has antioxidant, anticarcinogen, antibacterial, antidiabetic, anti-obesity, antiarthritic, antiviral and diuretic activities [14, 15]. SG is a promising mineral medicine with antioxidant, antiviral, and immunity-enhancement properties [16]. Similarly, SJ, GC, and DZ have been reported to show pharmacological activities of anti-inflammatory, antioxidant, anti-atherogenic, and antibacterial [17, 18]. However, the mechanism of YBD in treating NS still remains unknown. So, we used bioinformatics and *in vivo* experiment to explore the effect of YBD in treating NS.

**Figure 1:**
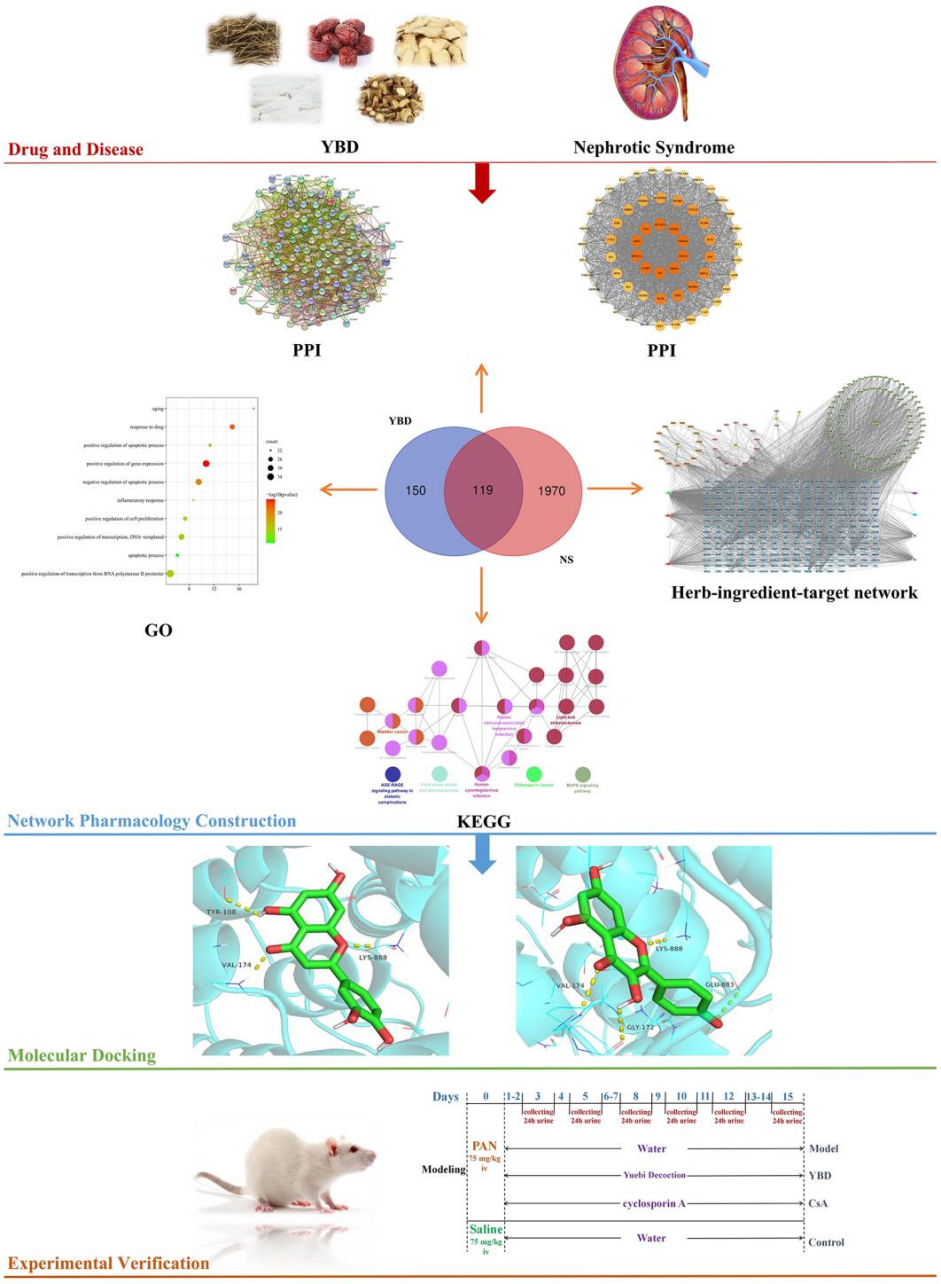
A comprehensive strategy diagram for the study of the mechanism of YBD in treating NS.

As is known, network pharmacology is regarded as an emerging and promising subject to explore the connections between active ingredients and diseases [19]. Through constructing “drug-ingredients-targets-pathways” network, network pharmacology effectively integrates computer technology and systems biology to reveal pharmacological mechanisms of TCM in treating related diseases [20]. In recent years, more and more researches related to TCM are conducted by network pharmacology [21, 22]. Here, active ingredients, targets of YBD in treating NS, and pathways were analyzed. Subsequently, *in vivo* experiment was conducted to verify the results (Figure 1). Our research gives the idea on treating NS and contributes to the clinical application of YBD in the future.

## MATERIALS AND METHODS

### Exploring of ingredients and potential targets of YBD

Five medicines from YBD, namely, Mahuang, Shigao, Shengjiang, Gancao, and Dazao were respectively inputted into the search box as keywords to retrieve in TCMSP (https://tcmsp-e.com/). Since the average value of oral bioavailability (OB) and drug-likeness (DL) from all molecules in DrugBank is 30% and 0.18 respectively, OB ≥30% and DL ≥0.18 are considered as significant parts in network pharmacology [23]. Then, TCMSP and SwissTargetPrediction were used to predict target proteins of active ingredients (http://www.swisstargetprediction.ch/). Finally, gene names were further mapped via UniProt database.

### Establishment of herb-ingredient-target (H-I-T) network

Cytoscape (version 3.9.1) was applied to establish a network between herbs, active ingredients of YBD and targets. As an open software for visualizing, Cytoscape software is widely applied in the field of network pharmacology [24].

### Screening of potential targets against NS

The targets were successfully obtained from GeneCards, PharmGKB and OMIM databases. The key word “nephrotic syndrome” was inputted as index words. The repeated targets corresponding to NS and YBD were deleted. Overlapping targets were successfully acquired by Venn diagram.

### Establishment of protein-protein interaction (PPI) network

STRING is an online and reliable database, which can be used to predict the interactions between proteins. Overlapping targets were inputted into STRING. In addition, disconnected genes in the network were hidden.

### Gene ontology (GO) and kyoto encyclopedia of genes and genome (KEGG) pathway enrichment analyses

GO contains biological process (BP), cellular component (CC), and molecular function (MF) [25, 26]. The Database for Annotation, Visualization and Integrated Discovery (DAVID) and ClueGO were applied to complete this part. The overlapping targets were entered into DAVID.

### Molecular docking simulation

To further explore connections between ingredients and targets, molecular docking was conducted. We chose five ingredients with the greatest number of overlapping targets as the ligands. Meanwhile, we selected five core targets as the receptors for verification. We searched for known ligands of protein receptors on the PDB to prepare for molecular docking. Firstly, 2D structures were acquired. Afterwards, 3D structures (mol2 format) with minimum energy of active ingredients were obtained from Chem3D software and converted into PDB format by PyMOL software as the ligands in molecular docking. Next, the PDB website was used to obtain the 3D structures of core targets. Then, PyMOL software was applied to delete water molecules, and those core targets were saved in PDB format as the receptors in molecular docking. Next, AutoDockTools was applied to convert small molecule ligands and receptors into PDBQT format. Finally, the binding was evaluated through Vina. −5 kcal/mol was set as a threshold affinity in this study.

### Experimental validation

### Experimental materials and preparation of YBD

The medicinal materials of YBD were provided by Yueyang Hospital. Cyclosporin A (CsA; Lot. H10960008) was purchased from North China Pharmaceutical Co., Ltd (China). Puromycin aminonucleoside (PAN; Lot. 108M4067V) was purchased from Sigma-Aldrich Co., Ltd. (USA). Podocin (PB9903) and nephrin (BM0676) antibodies were purchased from Boster (China). PPP3CA (YN2056), AKT1 (YN0514), STAT3 (YN0127), NFATC3 (YN0789), and TRPC6 (YN1223) antibodies were purchased from ImmunoWay Biotechnology Company (USA). According to the previous study [27], 660 g YBD were steeped for one hour in 5280 mL distilled water. After that, those dregs were filtered from the first extraction for the second extraction. Three times later, the concentration was 594 mg/mL and kept at 4°C for the further experiment.

### Chromatographic analysis of YBD

**Figure 2:**
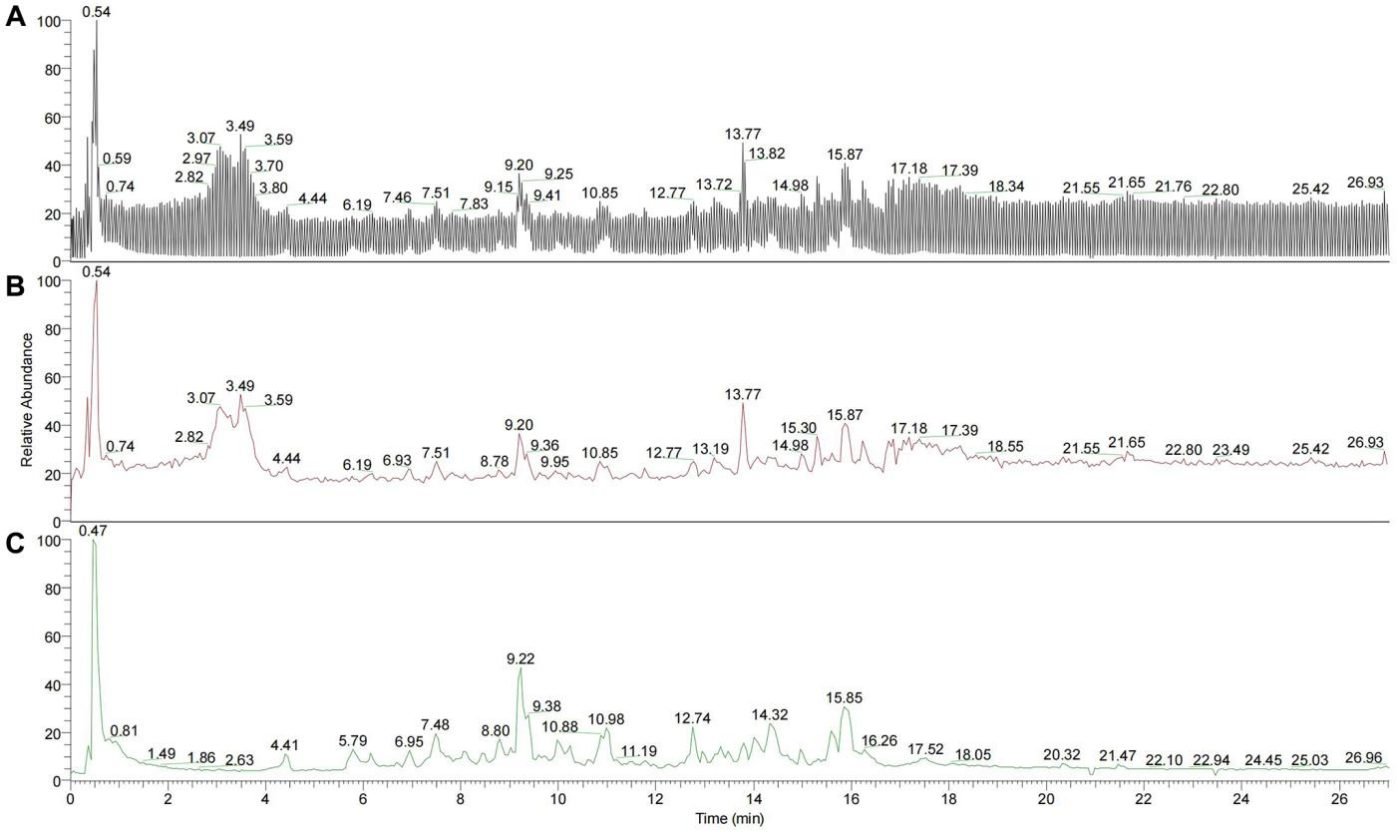
**Total ion flow diagram of YBD.** (**A**) Total negative and positive ion flow diagram superposition of YBD. (**B**) Total negative ion flow diagram of YBD. (**C**) Total positive ion flow diagram of YBD.

200 µL YBD (the concentration was 594 mg/mL) was used for ultrasonic dissolution. After centrifugation at temperature of 4°C with 20000 rpm for 10 min, 50 µL was extracted. Then, 450 µL precipitator was added to the supernatant. After centrifugation at temperature of 4°C with 20000 rpm for 10 min, 100 µL sample was applied for LC-MS/MS^2^. An UltiMate 3000 RS (Thermo Fisher Scientific) was applied to perform quantitative LC-MS/MS^2^ proteomic analysis. Finally, 215 compounds were acquired from YBD via chromatographic analysis (Figure 2), mainly including quercetin, kaempferol, luteolin, naringenin, formononetin, ephedrine hydrochloride, pseudoephedrine hydrochloride, 6-gingerol, isoliquiritigenin, catechin and glycyrrhetinic acid.

### Animal model establishment and drug administration

**Figure 3:**
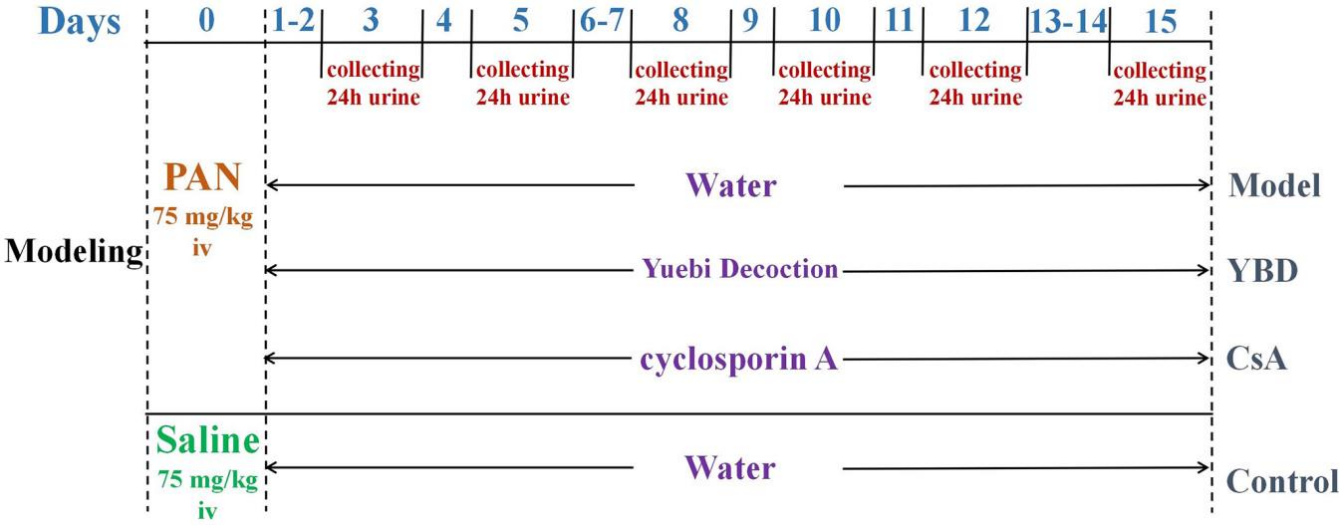
**The schematic representation of brief experimental schedule including modeling and treatment.** After acclimatization, 10 rats were randomly assigned to the Control group and intravenously injected with saline (75 mg/kg). Meanwhile, other rats were intravenously injected with PAN (75 mg/kg) to establish NS model on day 0. In the subsequent fifteen days, they were orally administered distilled water, YBD or CsA respectively.

Forty 5 to 6-week-old male Wistar rats weighing 160 ± 20 g were provided by Shanghai SLAC Laboratory Animal Co., Ltd in China (License NO. SCXK 2017–0005). Animal experiment was carried on SPF condition. Those rats were housed under conditions (23 ± 2°C, and 12 h/12 h light/dark cycle) with free access to standard rat chow and water [28]. The urine protein tests of the rats were negative. As shown in Figure 3, except 10 rats in the Control group, other rats were injected with PAN to establish NS model [29]. PAN has non-immune renal damaging effects and can induce the development of podocyte injury. It can cause the extensive fusion of foot processes, and lead to local cells stripped and exposed from the glomerular basement membrane (GBM). Rats in other groups received a single intravenous injection of PAN (75 mg/kg, dissolved in saline). Afterwards, those successful model were divided into three groups (each group includes 10 rats). The 24 h proteinuria was detected to ensure that there was no intergroup difference before treatment. Since calcineurin inhibitors (CNIs) served as a common strategy in treating NS, we chose CsA as the positive drug. For example, YBD was composed of five Chinese medicinal materials in Table 1, and it was taken 66 g daily for a patient with 70 kg weight in clinical. According to the equivalent dose of human and rats, the dose of rats = 66000 mg/kg/d × 0.018/0.2 kg, the same method was used to calculate the dose of CsA [30]. Drug treatments of each group were performed once daily for 15 days.

**Table 1:** Constituents of YBD.

**Number**	**Medicinal materials**	**Pharmaceutical name**	**Part used**	**Proportion**
1	Ma Huang	Ephedra sinica Stapf	stem	6
2	Shi Gao	Gypsum Fibrosum	mineral	8
3	Sheng Jiang	Zingiber officinale Rosc	rhizome	3
4	Gan Cao	Glycyrrhiza uralensis Fisch	radix	2
5	Da Zao	Ziziphus jujuba Mill	fruit	3

### Measurement of urinary protein, blood pressure, and serum biochemical analysis

At the end of the 3th, 5th, 8th, 10th, 12th and 15th day, the 24 h urine specimens were collected. Before the treatment and after 15 days of medicine treatment, the arterial pressure on the tails of rats was measured by noninvasive blood pressure collection system (BP-98A). Each rat was measured 3 times and the average level of blood pressure was calculated. When measuring the blood pressure, we waited 3 minutes for the rats to calm down. All the rats were sacrificed after the last treatment. Blood samples were obtained after the rats were anesthetized with pentobarbital sodium [31].

### F-actin cytoskeleton staining

Antigen retrieval was completed by Ethylene Diamine Tetraacetic Acid (EDTA) for kidney sections from different groups. Then, those sections were incubated with primary anti-F-actin. Afterwards, they were washed 3 times by PBS. Fifty minutes later, Actin-Tracker Green and DAPI were used to stain cell cytoskeleton and nucleus, respectively [32]. Under the microscope, F-actin was shown green by the tracer and the nucleus was stained blue by DAPI. Finally, Image Pro PLus 6.0 was used to analysis images. Colorful fluorescent images were first converted to black and white. Then, the same black color was chosen as a uniform standard for determining positivity, and the glomerular areas in images were segmented. Subsequently, the cumulative integrated optical density (IOD) and corresponding areas of the positive parts in images were acquired. IOD/AREA was calculated in each group.

### Western blotting analysis

Protein concentration was detected by BCA Protein Assay Kit. Then, total proteins were separated through 10% SDS-PAGE. The membranes were incubated with the antibodies of podocin (1:1000), nephrin (1:1000), PPP3CA (1:1000), STAT3 (1:1000), NFATC3 (1:1000), TRPC6 (1:1000), and AKT1 (1:1000). Finally, the membranes were washed by a multi-functional imaging system (Tanon-5200).

### Statistical analysis

SPSS 21.0 software was used for statistical analysis. Data were presented as mean ± SD. Comparisons among groups were made via ANOVA followed by Duncan’s test. Values with *p* < 0.05 were considered statistically significant.

### Data availability statement

The data used to support the findings of this study are available from the corresponding author upon request.

## RESULTS

### Ingredients of YBD and their potential targets

**Figure 4:**
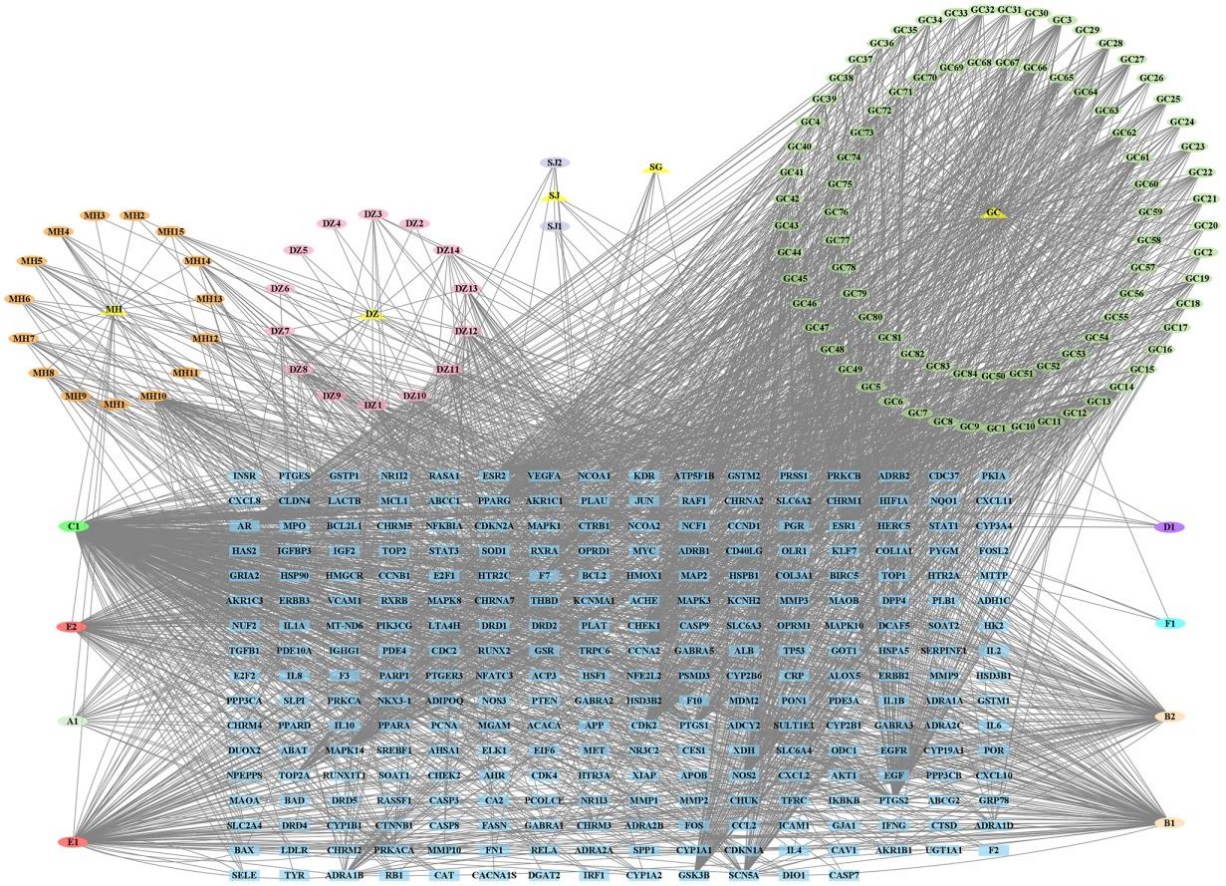
**Herb-ingredient-target network of YBD.** The yellow triangle nodes represented herbs, the green, orange, pink, gray, purple oval nodes represented different ingredients, and the blue rectangle nodes represented targets.

124 different ingredients from YBD were obtained from TCMSP and literature research in this study. There were 22 active ingredients from MH, 19 active ingredients from DZ, 5 active ingredients from SJ, 1 active ingredient from SG, and 88 active ingredients from GC. Detailed information was provided in Supplementary Table 1. 269 targets of the active ingredients were acquired through SwissTargetPrediction and TCMSP. Detailed information was provided in Supplementary Table 2. Obviously, quercetin, kaempferol, luteolin, naringenin, beta-sitosterol, 7-Methoxy-2-methyl isoflavone, stigmasterol, isorhamnetin, formononetin, and licochalcone A were top ten ingredients, which had greatest number of targets. Then, their CAS number, and chemical structure were provided in Table 2. Finally, in the network, there were 397 nodes (5 herbs, 124 active ingredients, and 269 targets) and 2570 edges (Figure 4).

**Table 2:** Information table of top ten active ingredients of YBD.

**CAS number**	**Ingredient name**	**Chemical structure**	**Molecular formula**	**Molecular weight**
117-39-5	Quercetin	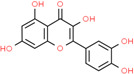	C_15_H_10_O_7_	302.24
520-18-3	Kaempferol	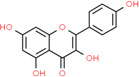	C_15_H_10_O_6_	286.24
491-70-3	Luteolin	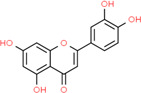	C_15_H_10_O_6_	286.24
480-41-1	Naringenin	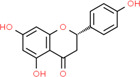	C_15_H_12_O_5_	272.25
83-46-5	Beta-sitosterol	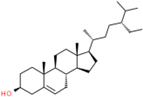	C_29_H_50_O	414.71
19725-44-1	7-Methoxy-2-methyl isoflavone	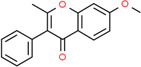	C_17_H_14_O_3_	266.31
83-48-7	Stigmasterol	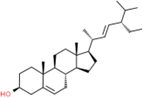	C_29_H_48_O	412.69
480-19-3	Isorhamnetin	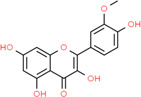	C_16_H_12_O_7_	316.28
485-72-3	Formononetin	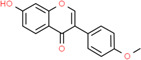	C_16_H_12_O_4_	268.28
58749-22-7	Licochalcone A	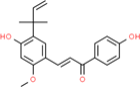	C_21_H_22_O_4_	338.43

### Screening of overlapping targets

**Figure 5:**
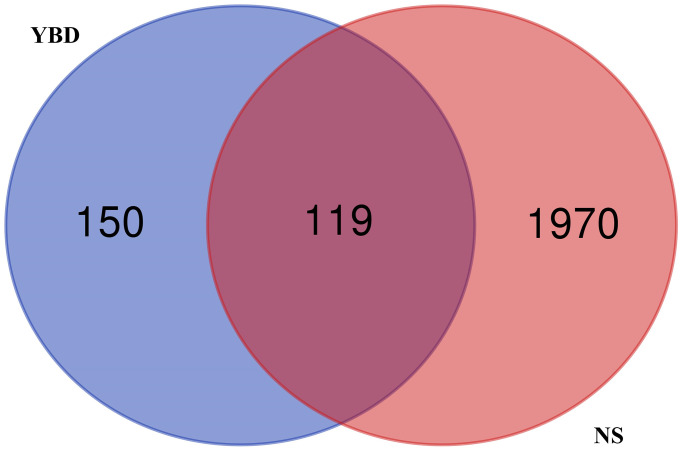
**Venn diagram of overlapping targets on YBD and NS.** The blue circle represented the targets of YBD, and the red circle represented the targets of NS. The part of the two intersecting circles represented the overlapping targets.

2089 NS-related targets were acquired through the public databases. Besides, 119 overlapping genes of between YBD and NS were acquired by a Venn diagram, which were regarded as potential therapeutic targets of YBD against NS (Figure 5). Detailed information was provided in Supplementary Table 3.

### PPI network and core targets

**Figure 6:**
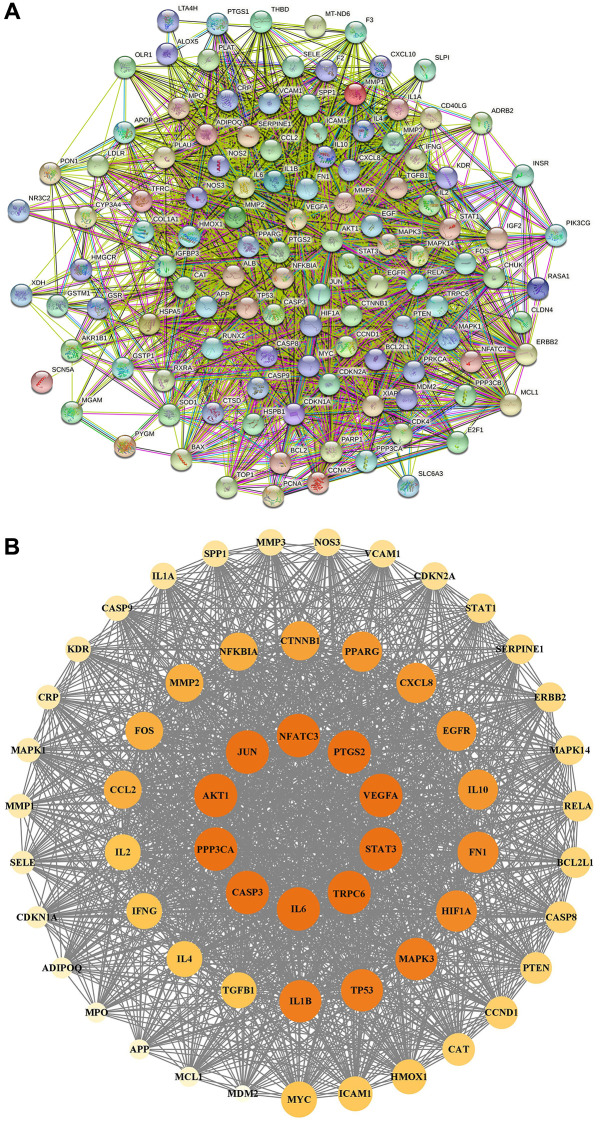
**PPI network construction.** (**A**) PPI network of potential therapeutic targets for YBD against NS through STRING database. (**B**) PPI network of core targets for YBD against NS through Cytoscape software. Colors from faint yellow to deep yellow are proportional to the degrees of nodes.

In Figure 6A, the PPI network included 118 nodes and 2595 edges. In Figure 6B, 59 core targets were acquired with value greater than 44.0. After putting 59 core targets into Cytoscape, we found a majority of them were related to podocyte injury, and the top ten targets were AKT1, STAT3, TRPC6, CASP3, JUN, PPP3CA, IL6, PTGS2, VEGFA, and NFATC3.

### GO and KEGG analysis

**Figure 7:**
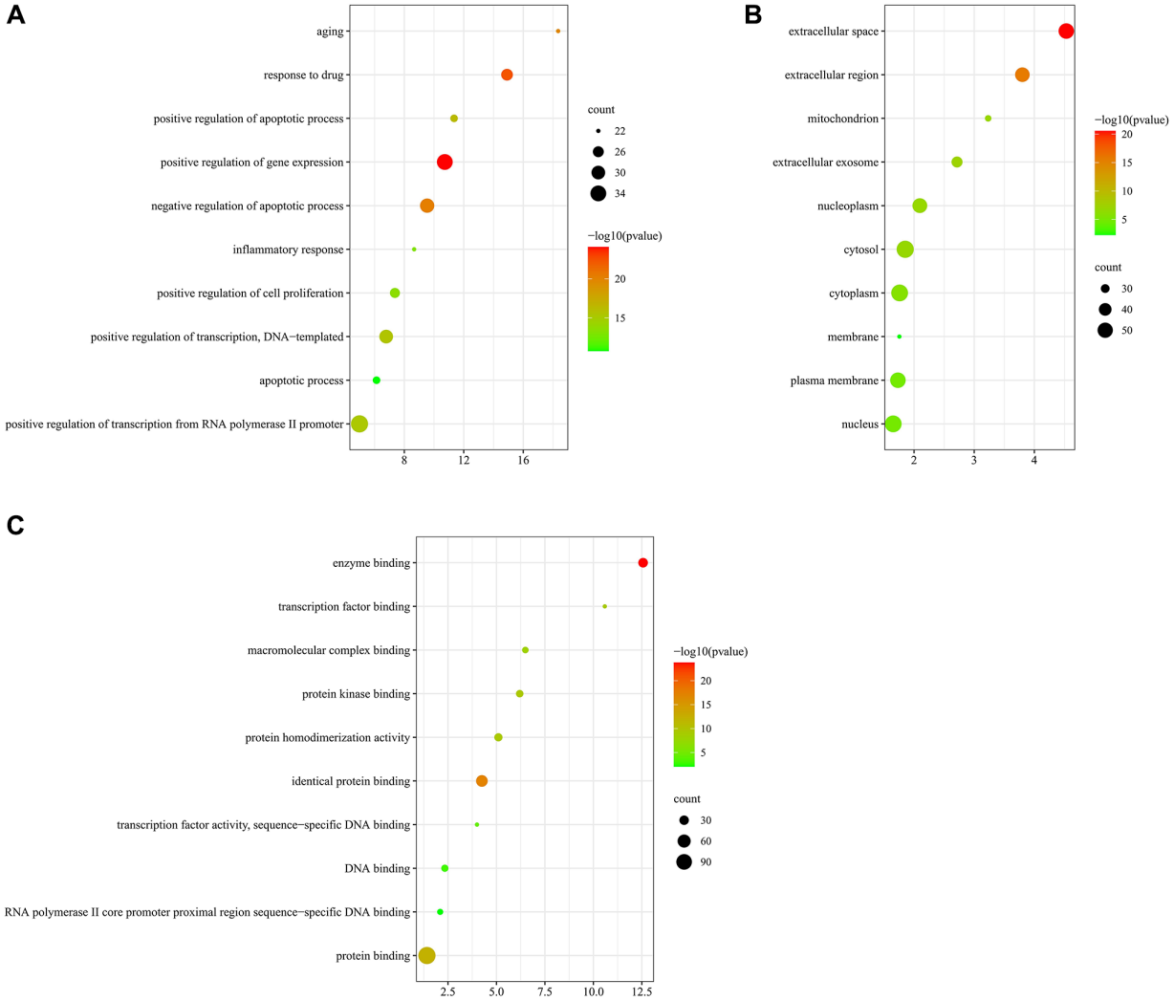
**GO enrichment analysis of 119 overlapping targets by DAVID database.** (**A**) the top 10 enriched terms in BP. (**B**) The top 10 enriched terms in CC. (**C**) The top 10 enriched terms in MF.

GO indicated that those targets were enriched in 513 BP, 89 CC, and 114 MF. In the BP ontology, the targets primarily associated with positive regulation of apoptotic process, apoptotic process, inflammatory response, aging (Figure 7A). In the CC ontology, the targets located mainly in cytosol, cytoplasm, nucleus, mitochondrion, and membrane (Figure 7B). In the MF ontology, it could be seen that the targets were mainly involved in protein homodimerization activity, protein kinase binding, macromolecular complex binding, and transcription factor activity (Figure 7C). Detailed information on GO enrichment analysis was performed in Supplementary Table 4.

**Figure 8:**
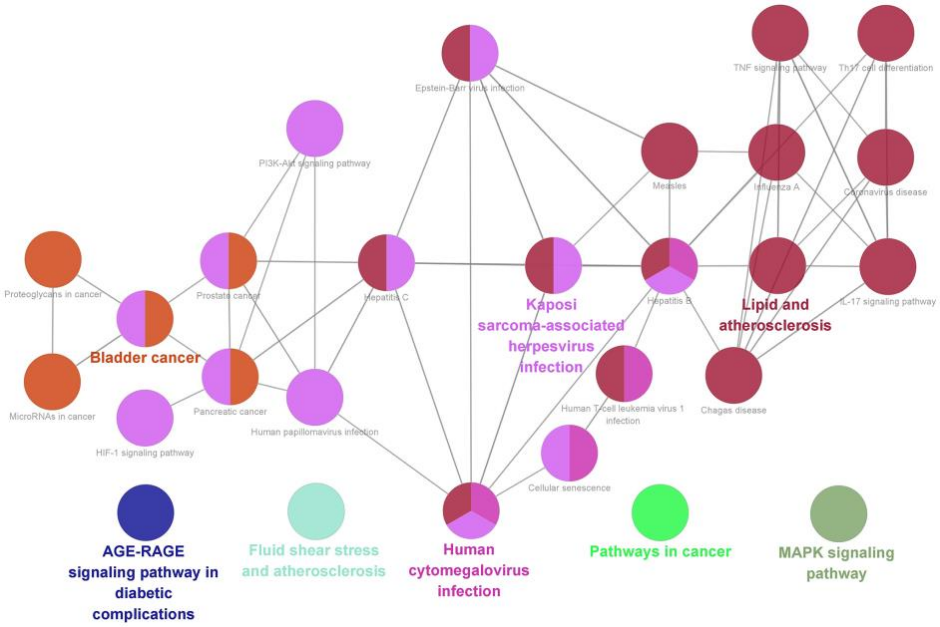
KEGG pathway analysis of 119 overlapping targets through ClueGO.

Finally, we acquired 27 representative pathways as shown in Figure 8. Those significant pathways could be divided into 8 categories, including AGE-RAGE signaling pathway in diabetic complications, MAPK signaling pathway, Fluid shear stress and atherosclerosis, Human cytomegalovirus infection, and Pathways in cancer.

### Molecular docking

**Figure 9:**
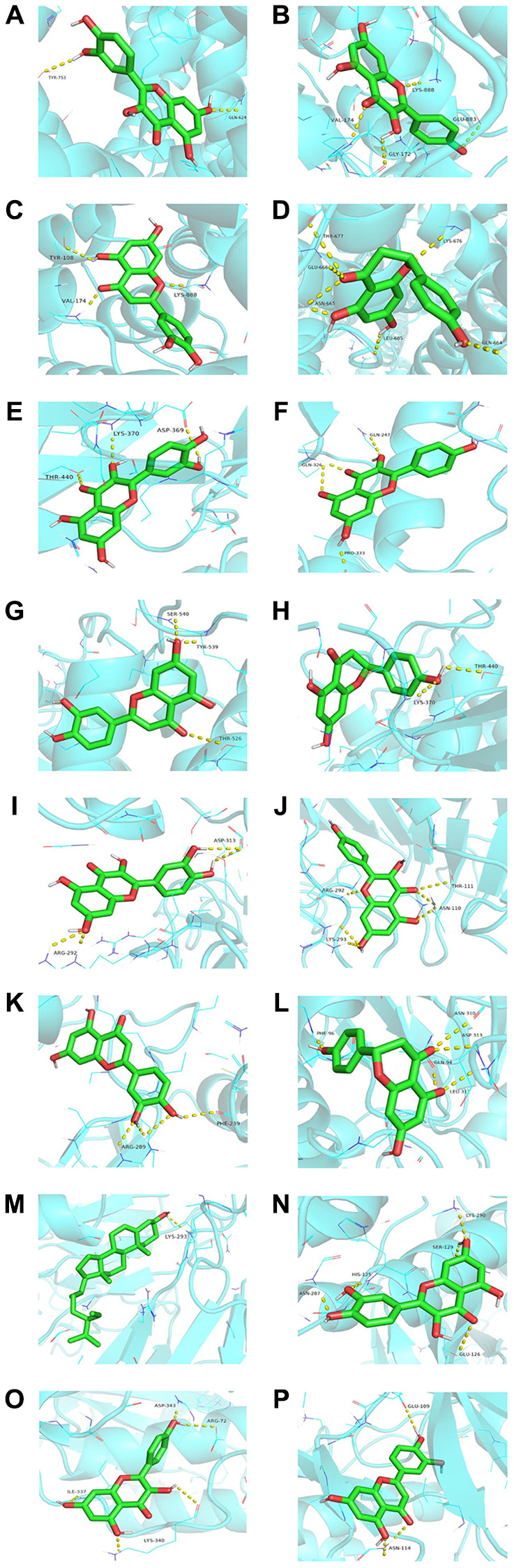
**Molecular docking of representative ingredients and targets (the affinity energy is less than or equal to −7.0 kcal/mol).** (**A**) Quercetin acts on TRPC6. (**B**) Kaempferol acts on TRPC6. (**C**) Luteolin acts on TRPC6. (**D**) Naringenin acts on TRPC6. (**E**) Quercetin acts on STAT3. (**F**) Kaempferol acts on STAT3. (**G**) Luteolin acts on STAT3. (**H**) Naringenin acts on STAT3. (**I**) Quercetin acts on PPP3CA. (**J**) Kaempferol acts on PPP3CA. (**K**) Luteolin acts on PPP3CA. (**L**) Naringenin acts on PPP3CA. (**M**) Beta-sitosterol acts on PPP3CA. (**N**) Quercetin acts on NFATC3. (**O**) Kaempferol acts on NFATC3. (**P**) Luteolin acts on NFATC3. The molecule was represented in a ball-stick model with atoms C and O in green and red, respectively.

Quercetin, kaempferol, luteolin, naringenin, and beta-sitosterol were regarded as ligands. Then, we selected AKT1, TRPC6, STAT3, PPP3CA, and NFATC3 as the protein receptors because they belonged to the top ten targets. Meanwhile, they were involved in development of podocyte injury and became critical targets in the treatment of NS through participating in many key biological processes including molecular trafficking, receptor activation, and signal transduction. The detailed information on the known ligands of AKT1, TRPC6, STAT3, PPP3CA, and NFATC3 were performed in Supplementary Table 5. In Table 3, quercetin owned the best affinity of −8.0 with TRPC6 and −7.8 with NFATC3. Kaempferol owned the best affinity of −8.1 with TRPC6 and −7.7 with PPP3CA. Luteolin owned the best affinity of −8.2 with TRPC6, −7.6 with STAT3, and −7.6 with PPP3CA. Naringenin owned the best affinity of −7.5 with TRPC6 and −7.4 with PPP3CA. Beta-sitosterol owned the best affinity of −7.0 with PPP3CA and −6.8 with TRPC6. Compared with affinity of the known ligand and its corresponding target, it showed that YBD had strong effects on its targets (Figure 9).

**Table 3:** Molecular docking scores of main active ingredients and targets.

**Group**	**AKT1**	**TRPC6**	**STAT3**	**PPP3CA**	**NFATC3**
Quercetin	−5.7	−8.0	−7.4	−7.7	−7.8
Kaempferol	−6.1	−8.1	−7.4	−7.7	−7.0
Luteolin	−6.2	−8.2	−7.6	−7.6	−7.3
Naringenin	−6.2	−7.5	−7.0	−7.4	−6.8
Beta-sitosterol	−5.0	−6.8	−6.4	−7.0	−5.0
Positive control	−6.2	−8.0	−7.1	−7.8	−8.2

### Verification of the effect of YBD on NS *in vivo*

### Effects of YBD on proteinuria, urine volume, and blood pressure in NS rats

**Figure 10:**
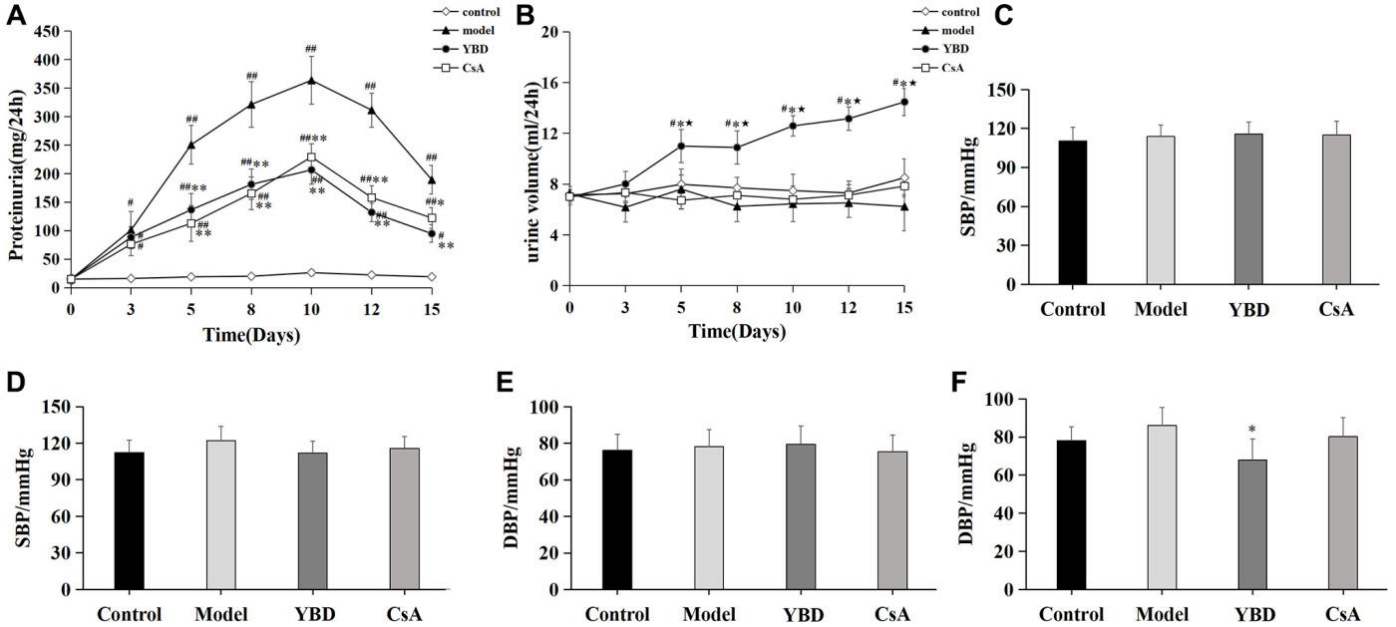
**Effects of YBD on proteinuria, urine volume, and blood pressure in NS rats induced by PAN.** (**A**) Effects of YBD on 24 h urinary protein excretion at various time points. (**B**) Effects of YBD on 24 h urine volume at various time points. (**C**) Systolic blood pressure (SBP) of rats before the treatment. (**D**) SBP of rats after the treatment. (**E**) Diastolic blood pressure (DBP) of rats before the treatment. (**F**) DBP of rats after the treatment. Data were expressed as mean ± SD, *n* = 10. Vertical bars represent the standard deviation. ^#^*p* < 0.05 and ^##^*p* < 0.01 versus control group; ^*^p < 0.05 and ^**^p < 0.01 versus model group; ^*^p < 0.05 versus CsA group.

We detected 24 h urine protein and urine volume at various time points, and found that proteinuria levels in the Model group peaked on day 10. However, YBD group exhibited a significant decline in 24 h proteinuria compared with the Model group from day 5 to day 15 (*p* < 0.01). While, CsA produced similar results compared with YBD (Figure 10A). Besides, we also found that YBD had an effect on urine volume. YBD significantly increased 24 h urine volume compared with the Control group from day 5 to day 15 (*p* < 0.05) (Figure 10B). To observe the effect of YBD on blood pressure, we compared the changes of SBP and DBP between each group. Before the treatment, no difference was exhibited on the level of SBP and DBP between the four groups (Figure 10C, 10E). After the treatment, it was found that DBP of rats treated with YBD decreased compared with the Model group (*p* < 0.05) (Figure 10F). Besides, SBP of rats in the YBD group decreased compared with the Model group. However, no difference was found on the level of SBP between the two groups (Figure 10D).

### Effects of YBD on ALB, BUN, SCr, SUA and serum lipid

**Figure 11:**
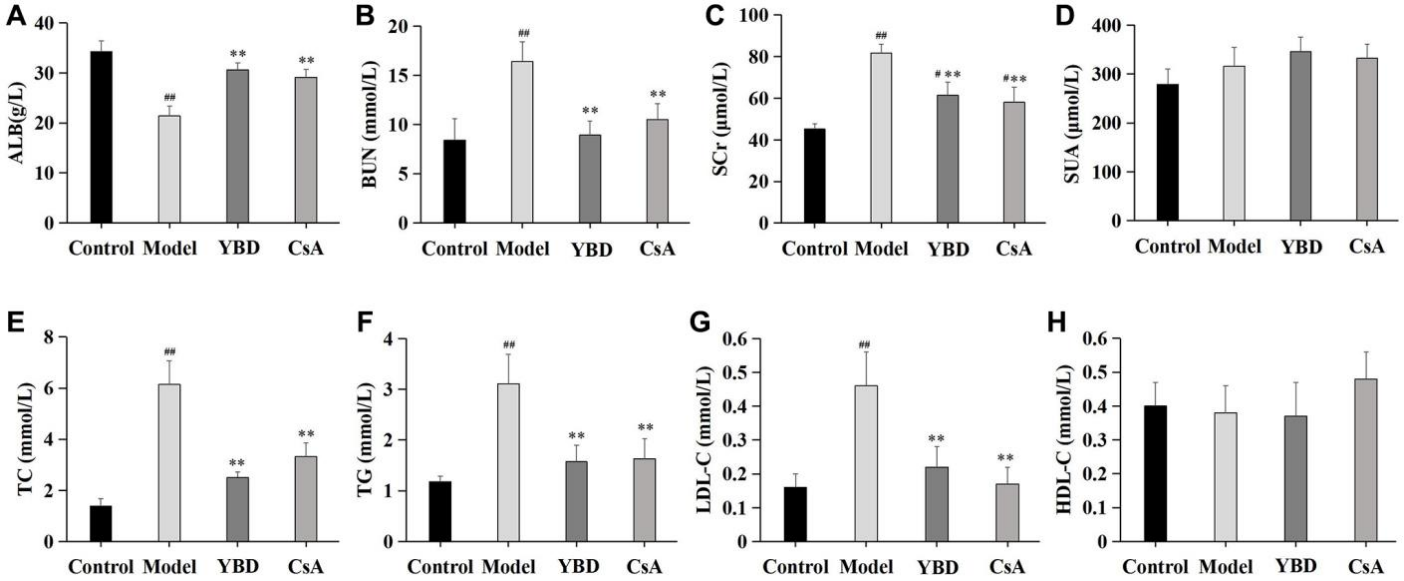
**Effects of YBD on albumin (ALB), renal function, and serum lipid in NS rats induced by PAN.** (**A**) The indexes of ALB in the serum. (**B**–**D**) The serum levels of renal function indicators (BUN, SCr, and SUA). (**E**–**H**) The serum lipid levels (TC, TG, LDL-C, and HDL-C) of each group. Data were expressed as mean ± SD, *n* = 10. Vertical bars represent the standard deviation. ^#^*p* < 0.05 and ^##^*p* < 0.01 versus control group; ^*^p < 0.05 and ^**^p < 0.01 versus model group.

As a remarkable symptom in NS, hypoalbuminemia was extremely showed in PAN-induced rats. Model group exhibited an decreased level of ALB when compared with Control group (*p* < 0.01). Both YBD and CsA groups had significantly increased the level of ALB compared with Model group (*p* < 0.01) (Figure 11A). YBD could improve renal function to some extent (Figure 11B–11D). The levels of BUN and SCr increased in Model group compared with Control group (*p* < 0.01). The levels of SCr and BUN decreased in YBD administration group compared with Model group (*p* < 0.01). TC, TG and LDL-C increased in Model group compared with Control group (*p* < 0.01). YBD could significantly decrease TC, TG and LDL-C compared with Model group (*p* < 0.01) (Figure 11E–11G)). While, no difference was exhibited on HDL-C between four groups (Figure 11H).

### Effects of YBD on histopathological changes in NS rats

**Figure 12:**
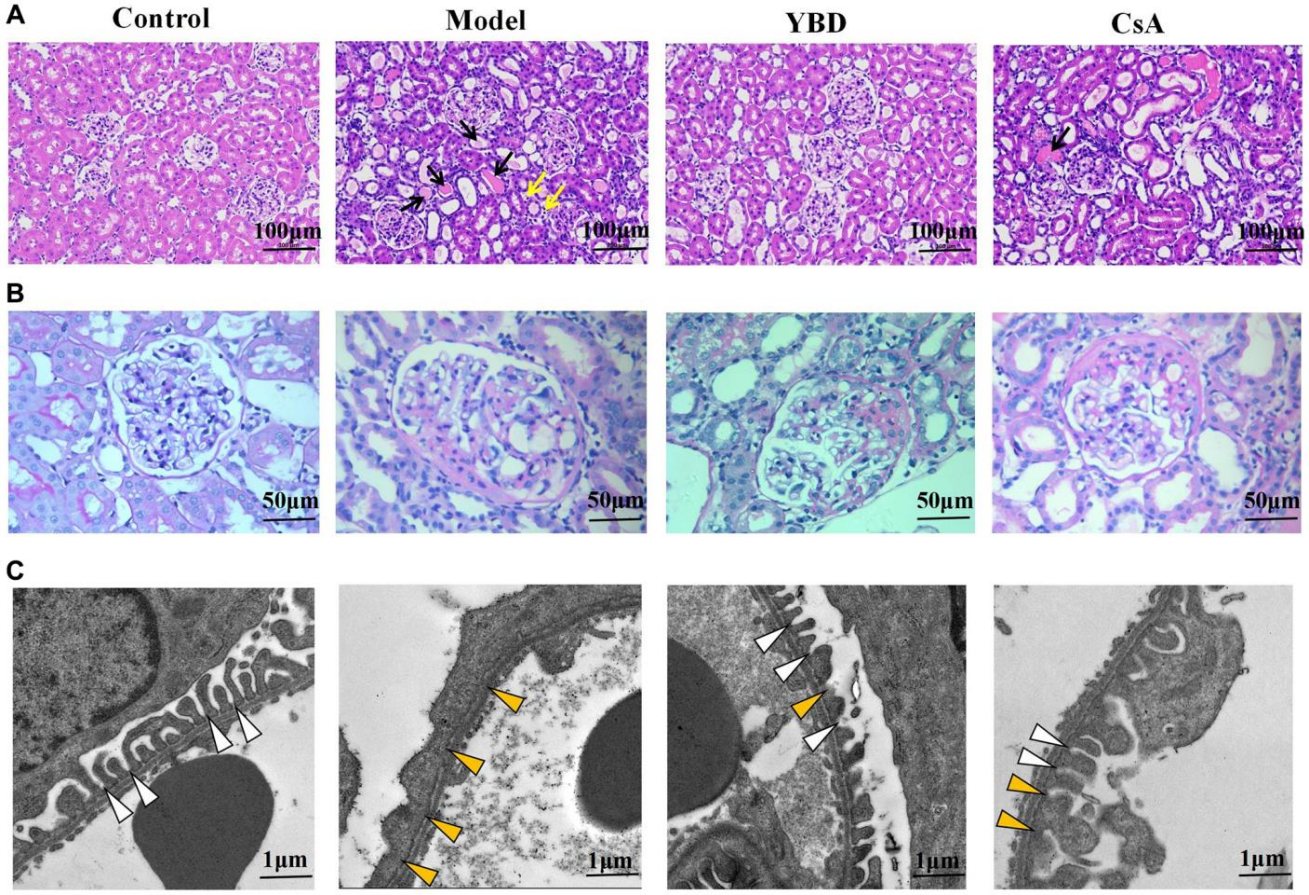
**Effects of YBD on renal histopathology in NS rats.** (**A**) Morphological observations with HE-stained in renal tissue of the four different groups (magnification×200). Protein casts were indicated by black arrows and inflammatory cell infiltration was indicated by yellow arrows. (**B**) Morphological observations with PAS-stained in renal tissue of the four different groups (magnification × 400). (**C**) Representative images of podocyte foot processes observed by transmission electron microscopy in different groups. The magnification of transmission electron microscopy was 5000. The normal structure of foot processes was marked by white arrow head, the effacement and fusion of foot processes were marked by yellow arrow head.

Through light microscopy, there were no significant histopathologic abnormalities in the glomerular structures among all groups. Model group exhibited abundant protein exudation in renal tubular lumens and inflammatory cell infiltration. However, YBD could attenuate the pathological damage through reducing protein cast formation and inflammatory cell (Figure 12A, 12B)). The transmission electron microscopy showed more clear morphological ultrastructure changes of podocyte (Figure 12C). Normal podocyte architecture could be seen in the Control group. However, injection of PAN evidently caused the massive effacement and extensive fusion of podocyte foot processes in the Model group. When treated with YBD, the effacement and fusion of foot processes significantly decreased.

### Effects of YBD on marker proteins of podocyte injury in NS rats

**Figure 13:**
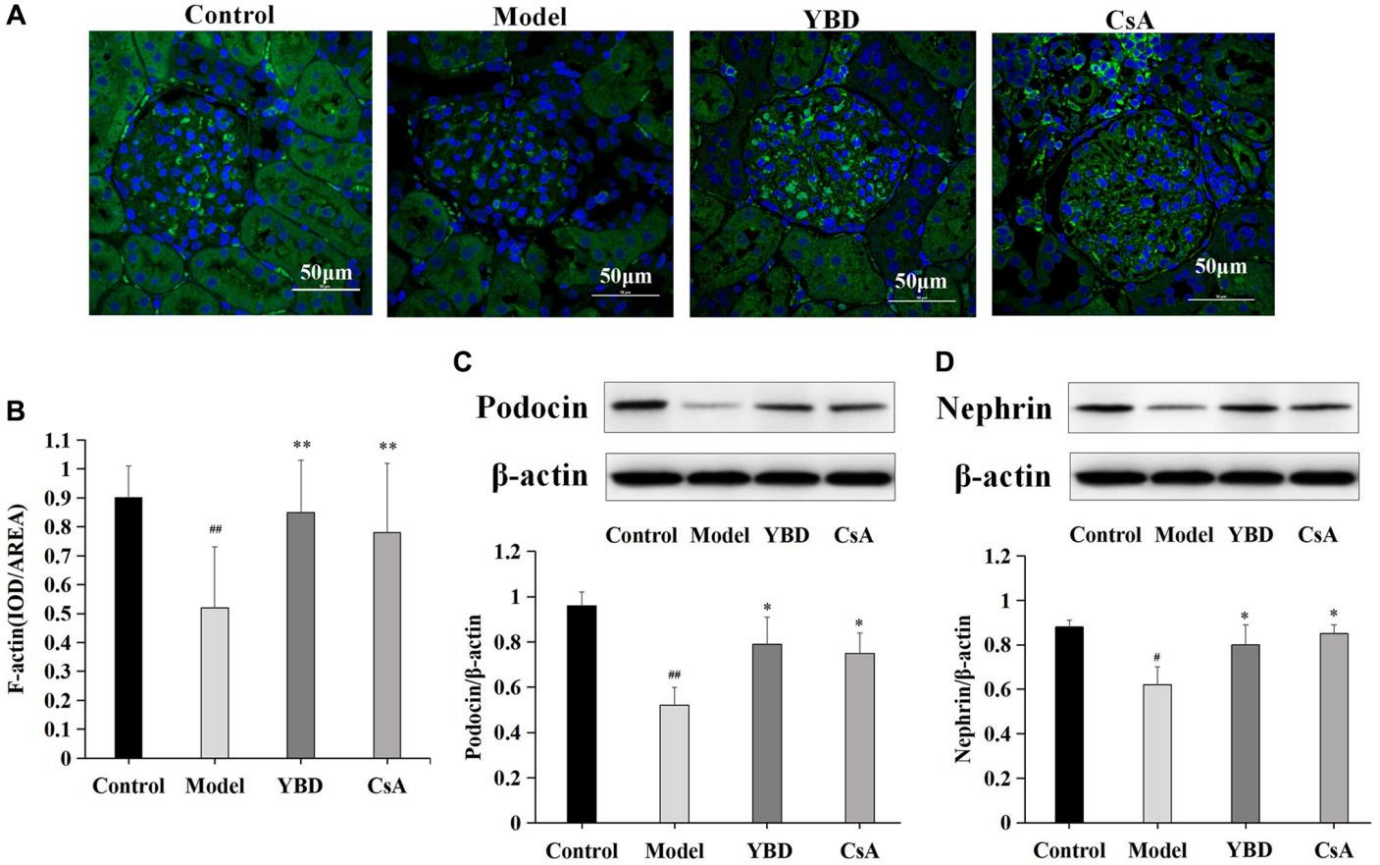
(**A**) F-actin in renal cortex tissues from different groups was shown by confocal microscopy (magnification × 600). Green staining showed positive expression of F-actin, and blue staining showed positive expression of cell nucleus. (**B**) The area of F-actin in different groups. (**C**, **D**) Western blotting analysis for the protein expression levels of podocin and nephrin in renal cortex tissues from different groups. As an internal control respectively, β-actin was used to calculate the quantification of protein. Data were expressed as mean ± SD, *n* = 6. Vertical bars represent the standard deviation. ^#^*p* < 0.05 and ^##^*p* < 0.01 versus control group; ^*^p < 0.05 and ^**^p < 0.01 versus model group.

We observed that PAN destroyed the amount and structure of F-actin stress fibers compared with Control group (*p* < 0.01). YBD could restore the amount of F-actin stress fibers and their structure prominently compared with Model group (*p* < 0.01) (Figure 13A, 13B). The decreased expression of nephrin or podocin is a hallmark of podocyte injury [33–35]. Western blotting was performed in this study to detect the expressions of nephrin and podocin. The expressions of podocin and nephrin markedly decreased in Model group compared with Control group. However, YBD could restore the low expressions of podocin and nephrin compared with Model group (*p* < 0.05) (Figure 13C, 13D).

### Effects of YBD on the expression levels of core targets

**Figure 14:**
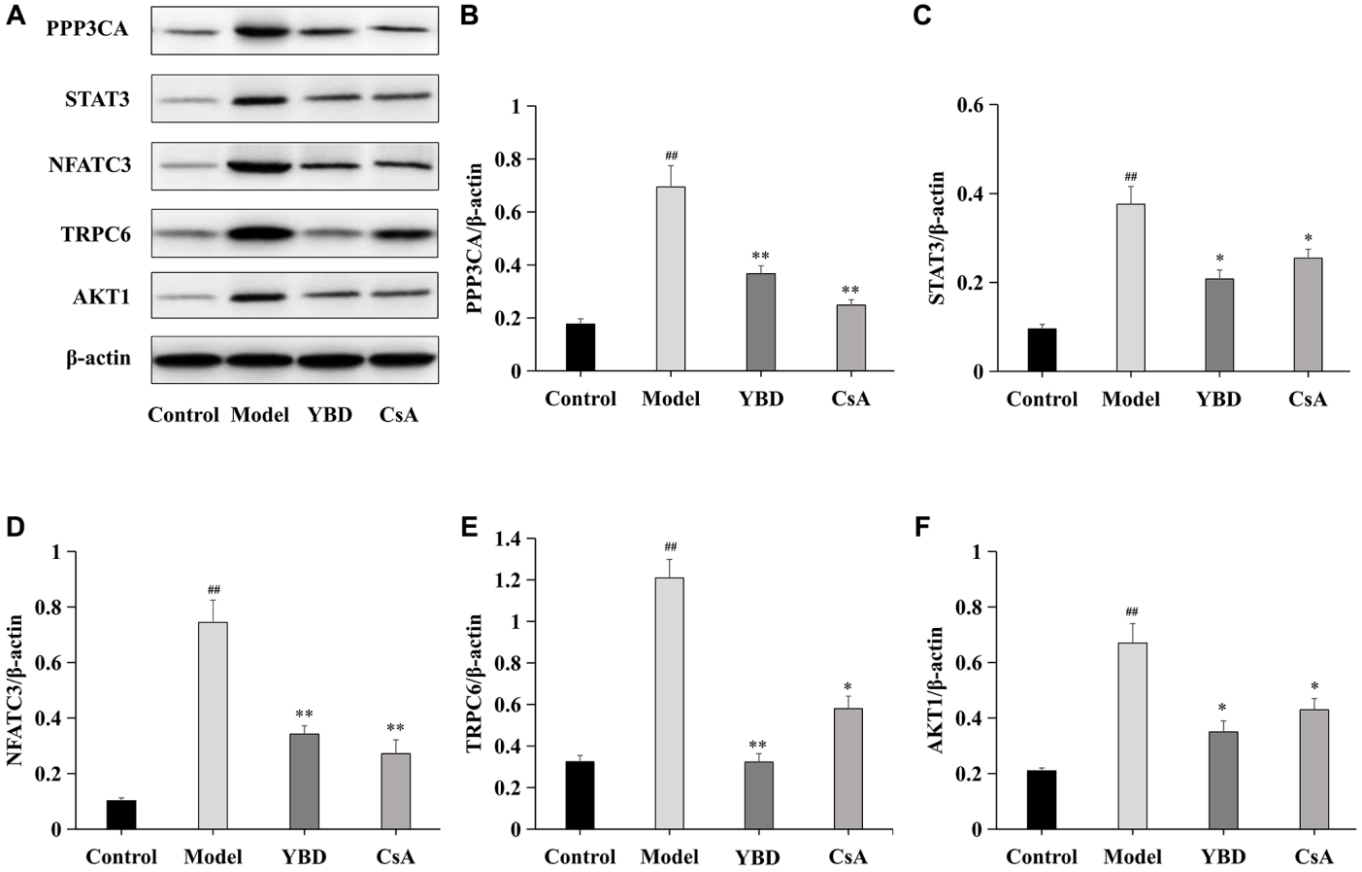
**Effects of YBD on the expressions of core targets in NS rats induced by PAN.** (**A**–**F**) The relative protein expressions of PPP3CA, STAT3, NFATC3, TRPC6, and AKT1 were measured by Western blotting. As an internal control respectively, β-actin was used to calculate the quantification of protein. Data were expressed as mean ± SD, *n* = 6. Vertical bars represent the standard deviation. ^#^*p* < 0.05 and ^##^*p* < 0.01 versus control group; ^*^p < 0.05 and ^**^p < 0.01 versus model group.

Five core targets (PPP3CA, STAT3, NFATC3, TRPC6, and AKT1) were collected as therapeutic targets of YBD. We examined the protein expressions of PPP3CA, STAT3, NFATC3, TRPC6, and AKT1 in renal tissues (Figure 14). Rats in Model group exhibited higher expressions of PPP3CA, STAT3, NFATC3, TRPC6, and AKT1 when compared with Control group (*p* < 0.01). However, after the treatment of YBD, PPP3CA, NFATC3, and TRPC6 in YBD group greatly decreased compared with Model group (*p* < 0.01). YBD could decrease high expressions of STAT3 and AKT1 compared with Model group (*p* < 0.05). Obviously, these indicated that YBD had protective effects on NS by improving podocyte injury.

## DISCUSSION

Proteinuria is an important marker of renal progression and acts as a key point in the clinical treatment. Recently, podocyte injury has gained acceptance as a key target leading to NS [36–38]. The highly differentiated polarized cell has a main body which bulges into the urinary space. In addition, podocyte injury is also regarded as an independent risk factor for the progression of renal diseases [39]. So, improving injured podocyte is crucial to the successful treatment of NS. Although corticosteroid and other immunosuppressive drugs have significantly reduced the risk of NS, it still remains unacceptably high morbidity in affected patient populations [40]. So, over the last two decades, numerous studies have been performed to study mechanisms of TCM in treating NS. YBD is a famous compound formula recorded in “Treatise on Febrile and Miscellaneous Diseases”, which has been applied in treating various forms of diseases related to edema and dysuria for years. Recently, we tried to use YBD in the treatment of NS and got a satisfied therapeutic effect. However, the multicomponent and multitarget characteristics, and underlying mechanism of YBD remains unknown.

Network pharmacology is a thriving interdisciplinary science and technology [41]. Through network pharmacology, 124 active ingredients and 119 overlapping targets were identified. The top ten were screened out, such as quercetin, kaempferol, luteolin, naringenin, beta-sitosterol, etc., As one of most important bioflavonoids, quercetin has positive effects on the biological processes and human health [42]. Quercetin can be extracted from MH, DZ, and GC, which could attenuate the level of podocyte apoptosis, and reduce the expression of pro-apoptotic protein Bax [43]. Kaempferol, a highly purified flavonoid active monomer, can been extracted from many edible plants and TCM, including MH and GC [44]. It has been demonstrated that kaempferol can reduce podocyte apoptosis and improve proteinuria, which is achieved by their modulation on M1/M2 polarization and the lowering effects on levels of IL-1β and TNF-α [45]. Luteolin is also the important ingredient of MH. It has a wide range of biological effects, including anti-oxidative, and anti-inflammatory properties [46]. Luteolin has been reported in combination with prednisone for the treatment of NS [47], and its underlying mechanism is connected with the inhibition of podocyte injury by regulating NLRP3 inflammasome [48].

AKT1, STAT3, TRPC6, CASP3, JUN, PPP3CA, IL6, PTGS2, VEGFA, and NFATC3 were the top 10 targets. Previous researches have confirmed that some of these targets were closely related to the pathogenic process of NS via participating in podocyte injury [49]. For example, as one of the most important components of TRPC family, TRPC6 has been regarded as the key target for the development of therapeutic agents to NS [50]. On mechanism, TRPC6 is known as a multiple transmembrane protein that mediates the release of cytosolic calcium [51]. The abnormal expression of TRPC6 is most commonly associated with podocyte injury and will accelerate the progression of NS. [52]. When TRPC6 is up-regulated, it would cause the excessive release of cytosolic calcium from podocyte [53]. With the entry of calcium, the expression of PPP3CA is promoted under the intervention of TRPC6 [54]. Afterwards, consequent translocation of NFATC3 to the nucleus happens, which further leads to the low expression levels of nephrin, podocin and F-actin [55]. Finally accompanied by fusion of podocyte foot processes, cytoskeleton injury and apoptosis of podocyte were found [56]. Abnormal activation of STATA3 results in podocyte injury and proteinuria [57, 58]. AKT1 is essential to maintain podocyte viability and function during the progression of NS, and through stimulating AKT1 phosphorylation, podocyte apoptosis can be inhibited [59].

Furthermore, GO showed that core targets functioned in the regulation of cell apoptotic process, including podocyte. It is precisely because of podocyte injury that leads to the damage of glomerular filtration barrier and gradually underlies the pathophysiology of NS [60, 61]. Therefore, it is believed that the efficacy of YBD in treating NS is associated with inhibition of podocyte injury. In KEGG pathway enrichment analysis, targets were enriched in AGE-RAGE signaling pathway, MAPK signaling pathway, and pathways in cancer. For example, AGE-RAGE plays a major role in pathophysiology of NS caused by diabetes. It has been reported that abnormal activation of AGE-RAGE stimulates NADPH oxidase-mediated reactive oxygen species production, leading to glomerular hypertrophy, podocyte injury, and mass proteinuria [62]. MAPK is closely associated with the disruption of cytoskeletal proteins (podocin and nephrin), endoplasmic reticulum stress activation, and apoptosis, which is an important pathway in treating NS [63].

Molecular docking is a significant method to predict the affinity [64]. It could be found that the energy of main ingredients to core proteins was no more than −5.0 kcal/mol. According to Figure 8, quercetin, kaempferol, and luteolin could closely bind to the various targets, such as AKT1, TRPC6, STAT3, PPP3CA, and NFATC3. Obviously, the best binding affinity (−8.2 kcal/mol) was observed between luteolin and TRPC6, which mainly depended on the hydrogen bond interaction with TYR-108, VAL-174, and LYS-888.

PAN-induced NS model is a representative animal model and has been used to study glomerular proteinuria [65, 66]. When the model was successfully established, the massive proteinuria and decreased level of ALB was observed. Luckily, direct protective effects of YBD were observed in PAN-induced rats, including reducing proteinuria, decreasing blood pressure, increasing urine volume and ALB, ameliorating the condition of renal function and dyslipidemia. Through transmission electron microscopy, we found that the massive effacement and extensive fusion of podocyte foot processes were ameliorated with the treatment of YBD. Moreover, reduced expressions of nephrin and podocin, as well as actin-associated protein F-actin, were found in the NS rats, which were alleviated after administration of YBD. Although a previous study has shown that YBD can effectively regulate water metabolism to reduce lung and kidney edema of severe acute pancreatitis rats via decreasing aquaporins expression [67]. Our results provided the evidence that YBD protected against podocyte injury in PAN-induced NS rats through improving the expressions of nephrin, podocin and F-actin. In addition, western blotting analysis showed that YBD could significantly inhibit the expressions of PPP3CA, STAT3, NFATC3, TRPC6, and AKT1 in renal tissues. When combined with network pharmacology, it indicates YBD can been applied in treating NS by targeting proteins associated with podocyte injury, including PPP3CA, STAT3, NFATC3, TRPC6, and AKT1.

Obviously, these experiment results demonstrated the therapeutic effect of YBD, and verified the prediction information obtained through bioinformatics. However, it mainly focused on NS caused by podocyte injury. As is known, NS contains a variety of pathological types, including MCD, FSGS, MN, and IgAN [68]. Among them, MCD, FSGS, MN are characterized by different degrees of podocyte injury, which are known as the podocytopathy [69]. In this study, we examined effects of YBD on PAN-induced nephrosis rats, a well-established model of podocyte injury and human NS [70]. So, we speculated that YBD may be effective in treating certain pathological types of NS, such as MCD, FSGS, and MN.

## CONCLUSIONS

In summary, this study firstly puts forward a comprehensive strategy that combines bioinformatics and animal experiment to study material basis of YBD and its possible mechanisms against NS. Firstly, the relevant ingredients and core targets of YBD in treating NS were searched. Then, key ingredients, core targets (AKT1, STAT3, TRPC6, CASP3, JUN, PPP3CA, IL6, PTGS2, VEGFA, and NFATC3), and pathways related to podocyte injury (AGE-RAGE, and MAPK) were predicted by network pharmacology. Besides, molecular docking was applied to prove that ingredients had good affinity with target proteins. Finally, *in vivo* experiment confirmed effects of YBD and revealed that mechanisms are related to the regulations of PPP3CA, STAT3, NFATC3, TRPC6, and AKT1. However, the therapeutic effects and molecular mechanisms related to podocyte injury of YBD and its main active ingredients need to be studied in the future.

## Supplementary Materials

Supplementary Tables 1-3 and 5

Supplementary Table 4
