# Supplementary material for: Exploring the therapeutic mechanism of Yuebi decoction on nephrotic syndrome based on network pharmacology and experimental study
Source: Aging (Albany NY). 2024 Sep 20;16(18):12623–50. doi: 10.18632/aging.206116 (PMC11466484; doi:10.18632/aging.206116)
Supplement: Supplementary Tables 1-3 and 5 [file aging-16-206116-s001.pdf]

SUPPLEMENTARY TABLES

Supplementary Table 1. A total of 124 different active ingredients from TCMSP and literature in YBD.

Ingredients	Herb	Symbol
(+)-Catechin	MH	A1
(+)-Leucocyanidin	MH	MH1
24-Ethylcholest-4-en-3-one	MH	MH2
Beta-sitosterol	MH	B1
Campest-5-en-3beta-ol	MH	MH3
Delphinidin	MH	MH4
Diosmetin	MH	MH5
Eriodictyol	MH	MH6
Genkwanin	MH	MH7
Herbacetin	MH	MH8
Kaempferol	MH	E1
Leucopelargonidin	MH	MH9
Luteolin	MH	MH10
Mandenol	MH	MH11
Naringenin	MH	E2
Pectolinarigenin	MH	MH12
Poriferast-5-en-3beta-ol	MH	D1
Quercetin	MH	C1
Resivit	MH	MH13
Stigmasterol	MH	B2
Taxifolin	MH	MH14
Truflex OBP	MH	MH15
stepharine	DZ	DZ1
zizyphus saponin I_qt	DZ	DZ2
coumestrol	DZ	DZ3
Daechuine S7	DZ	DZ4
Jujubasaponin V_qt	DZ	DZ5
Mauritine D	DZ	DZ6
berberine	DZ	DZ7
(S)-Coclaurine	DZ	DZ8
Mairin	DZ	F1
Stigmasterol	DZ	B2
beta-sitosterol	DZ	B1
Ruvoside_qt	DZ	DZ9
(+)-catechin	DZ	A1
Stepholidine	DZ	DZ10
Nuciferin	DZ	DZ11
Fumarine	DZ	DZ12
beta-carotene	DZ	DZ13
(−)-catechin	DZ	DZ14
quercetin	DZ	C1
beta-sitosterol	SJ	B1
6-methylgingediacetate2	SJ	SJ1
Stigmasterol	SJ	B2

poriferast-5-en-3beta-ol	SJ	D1
Dihydrocapsaicin	SJ	SJ2
CaSO4	SG	SG
quercetin	GC	C1
Mairin	GC	F1
Jaranol	GC	GC1
isorhamnetin	GC	GC2
sitosterol	GC	GC3
formononetin	GC	GC4
Calycosin	GC	GC5
kaempferol	GC	GC6
licochalcone a	GC	E1
Vestitol	GC	GC7
Inermine	GC	GC8
DFV	GC	GC9
Glycyrol	GC	GC10
Medicarpin	GC	GC11
Lupiwighteone	GC	GC12
7-Methoxy-2-methyl isoflavone	GC	GC13
naringenin	GC	GC14
(2S)-2-[4-hydroxy-3-(3-methylbut-2-enyl)phenyl]-8,8-dimethyl-2,3-dihydropyrano[2,3-f]chromen-4-one	GC	E2
euchrenone	GC	GC15
glyasperin B	GC	GC16
glyasperin F	GC	GC17
Glyasperin C	GC	GC18
Isotrifoliol	GC	GC19
(E)-1-(2,4-dihydroxyphenyl)-3-(2,2-dimethylchromen-6-yl)prop-2-en-1-one	GC	GC20
kanzonols W	GC	GC21
(2S)-6-(2,4-dihydroxyphenyl)-2-(2-hydroxypropan-2-yl)-4-methoxy-2,3-dihydrofuro[3,2-g]chromen-7-one	GC	GC22
Semilicoisoflavone B	GC	GC23
Glepidotin A	GC	GC24
Glepidotin B	GC	GC25
Phaseolinisoflavan	GC	GC26
Glypallichalcone	GC	GC27
8-(6-hydroxy-2-benzofuranyl)-2,2-dimethyl-5-chromenol	GC	GC28
Licochalcone B	GC	GC29
licochalcone G	GC	GC30
3-(2,4-dihydroxyphenyl)-8-(1,1-dimethylprop-2-enyl)-7-hydroxy-5-methoxy-coumarin	GC	GC31
Licoricone	GC	GC32
Gancaonin A	GC	GC33
Gancaonin B	GC	GC34
3-(3,4-dihydroxyphenyl)-5,7-dihydroxy-8-(3-methylbut-2-enyl)chromone	GC	GC35
5,7-dihydroxy-3-(4-methoxyphenyl)-8-(3-methylbut-2-enyl)chromone	GC	GC36
2-(3,4-dihydroxyphenyl)-5,7-dihydroxy-6-(3-methylbut-2-enyl)chromone	GC	GC37
Glycyrin	GC	GC38
Licocoumarone	GC	GC39
Licoisoflavone	GC	GC40
Licoisoflavone B	GC	GC41

licoisoflavanone	GC	GC42
shinpterocarpin	GC	GC43
(E)-3-[3,4-dihydroxy-5-(3-methylbut-2-enyl)phenyl]-1-(2,4-dihydroxyphenyl)prop-2-en-1-one	GC	GC44
liquiritin	GC	GC45
licopyranocoumarin	GC	GC46
Glyzaglabrin	GC	GC47
Glabridin	GC	GC48
Glabranin	GC	GC49
Glabrene	GC	GC50
Glabrone	GC	GC51
1,3-dihydroxy-9-methoxy-6-benzofurano[3,2-c]chromenone	GC	GC52
1,3-dihydroxy-8,9-dimethoxy-6-benzofurano[3,2-c]chromenone	GC	GC53
Eurycarpin A	GC	GC54
(−)-Medicocarpin	GC	GC55
Sigmoidin-B	GC	GC56
(2R)-7-hydroxy-2-(4-hydroxyphenyl)chroman-4-one	GC	GC57
(2S)-7-hydroxy-2-(4-hydroxyphenyl)-8-(3-methylbut-2-enyl)chroman-4-one	GC	GC58
Isoglycyrol	GC	GC59
Isolicoflavonol	GC	GC60
HMO	GC	GC61
1-Methoxyphaseollidin	GC	GC62
Quercetin der.	GC	GC63
3'-Hydroxy-4'-O-Methylglabridin	GC	GC64
3'-Methoxyglabridin	GC	GC65
2-[(3R)-8,8-dimethyl-3,4-dihydro-2H-pyrano[6,5-f]chromen-3-yl]-5-methoxyphenol	GC	GC66
Inflacoumarin A	GC	GC67
icos-5-enoic acid	GC	GC68
Kanzonol F	GC	GC69
6-prenylated eriodictyol	GC	GC70
7,2',4'-trihydroxy-5-methoxy-3-arylcoumarin	GC	GC71
7-Acetoxy-2-methylisoflavone	GC	GC72
8-prenylated eriodictyol	GC	GC73
gadelaidic acid	GC	GC74
Gancaonin G	GC	GC75
Gancaonin H	GC	GC76
Licoagrocarpin	GC	GC77
Glyasperins M	GC	GC78
Glycyrrhiza flavonol A	GC	GC79
Licoagroisoflavone	GC	GC80
Odoratin	GC	GC81
Phaseol	GC	GC82
Xambioona	GC	GC83
dehydroglyasperins C	GC	GC84

Supplementary Table 2. Targets information of different ingredients in YBD through SwissTargetPrediction and TCMSP.

PSMD3	target	HSPB1	target	RAF1	target	GABRA2	target	CHRNA7	target
MMP2	target	HMOX1	target	RASSF1	target	IKBKB	target	CHRNA2	target
HSPA5	target	HK2	target	RASA1	target	MAPK8	target	JUN	target
ACHE	target	NKX3-1	target	ERBB2	target	CHRM2	target	TGFB1	target
ACACA	target	HAS2	target	ERBB3	target	SLC6A2	target	F10	target
AHSA1	target	HIF1A	target	RB1	target	CA2	target	ADH1C	target
AKR1B1	target	CHUK	target	RXRA	target	CDK4	target	ADRA2A	target
MAOB	target	INSR	target	RUNX2	target	FOSL2	target	MAOA	target
AR	target	IGF2	target	CHEK2	target	STAT3	target	CTRB1	target
BAX	target	IGFBP3	target	PON1	target	HTR2A	target	DRD5	target
BCL2	target	ICAM1	target	STAT1	target	CHRM4	target	ADRA2B	target
ALOX5	target	IFNG	target	SCN5A	target	HTR3A	target	DRD2	target
AHR	target	IRF1	target	SLC2A4	target	ADRA1D	target	CYP2B6	target
BIRC5	target	IL1A	target	MMP3	target	IGHG1	target	CDC37	target
BCL2L1	target	IL1B	target	SOD1	target	CHRM3	target	PDE10A	target
ADRB2	target	IL10	target	THBD	target	OPRM1	target	ADRA2C	target
CASP3	target	IL2	target	F3	target	KDR	target	LACTB	target
CASP8	target	IL6	target	PLAT	target	OPRD1	target	DRD4	target
CASP9	target	CXCL8	target	E2F1	target	DRD1	target	HTR2C	target
CTSD	target	MMP1	target	E2F2	target	MAPK10	target	TOP2	target
CAV1	target	MMP9	target	RELA	target	CHRM5	target	PDE4	target
CCL2	target	MAPK1	target	PRSS1	target	ADRB1	target	CACNA1S	target
CD40LG	target	MYC	target	NFATC3	target	LTA4H	target	CYP2B1	target
TP53	target	MPO	target	DIO1	target	HMGCR	target	ALB	target
CLDN4	target	NQO1	target	PLAU	target	ABAT	target	CTNNB1	target
F7	target	POR	target	VCAM1	target	ADIPOQ	target	CASP7	target
COL1A1	target	NCF1	target	VEGFA	target	AKR1C1	target	MMP10	target
COL3A1	target	NFKBIA	target	PPP3CA	target	APOB	target	KLF7	target
CRP	target	NOS3	target	PGR	target	GOT1	target	GRP78	target
CXCL10	target	NFE2L2	target	CDK2	target	BAD	target	ABCG2	target
CXCL11	target	NCOA2	target	ESR2	target	CAT	target	CDC2	target
CXCL2	target	NR1I2	target	NOS2	target	CYP19A1	target	CDKN2A	target
CDKN1A	target	NR1I3	target	CHEK1	target	FASN	target	IL8	target
CYP1A1	target	ODC1	target	CCNA2	target	GSR	target	MGAM	target
CYP1A2	target	SPP1	target	ESR1	target	CES1	target	HERC5	target
CYP1B1	target	PPARG	target	GRIA2	target	LDLR	target	F2	target
CYP3A4	target	PPARA	target	PYGM	target	MTTP	target	PPP3CB	target
DCAF5	target	PPARD	target	GSK3B	target	MAPK3	target	ADCY2	target
DPP4	target	PTEN	target	MAPK14	target	ABCC1	target	APP	target
TOP1	target	PIK3CG	target	NCOA1	target	PLB1	target	XIAP	target
TOP2A	target	SERPINE1	target	OLR1	target	SOAT1	target	MDM2	target
DUOX2	target	PARP1	target	NR3C2	target	SOAT2	target	MET	target
EGFR	target	KCNH2	target	HSD3B1	target	SREBF1	target	MCL1	target
SELE	target	PCOLCE	target	HSD3B2	target	UGT1A1	target	NUF2	target
SULT1E1	target	EGF	target	ADRA1A	target	RXRB	target	PCNA	target
ELK1	target	PTGER3	target	ATP5F1B	target	KCNMA1	target	PTGES	target
EIF6	target	PTGS1	target	PKIA	target	TFRC	target	TYR	target
CCND1	target	PTGS2	target	IL4	target	FN1	target	XDH	target
CCNB1	target	ACP3	target	CHRM1	target	TRPC6	target	DGAT2	target
GABRA1	target	RUNX1T1	target	MT-ND6	target	PDE3A	target		
GJA1	target	PRKCA	target	SLC6A3	target	GABRA3	target		
GSTM1	target	PRKCB	target	SLC6A4	target	GABRA5	target		

GSTM2	target	FOS	target	AKR1C3	target	HSP90	target
GSTP1	target	NPEPPS	target	ADRA1B	target	MAP2	target
HSF1	target	AKT1	target	SLPI	target	PRKACA	target

Supplementary Table 3. Overlapping targets information of drug targets and disease targets.

MMP2
XDH
HSPB1
NOS2
COL1A1
PPP3CA
BCL2
CRP
GSTP1
FN1
EGF
IL1A
LDLR
SPP1
F2
XIAP
PTGS2
OLR1
CTNNB1
CCND1
CDK4
NFATC3
VEGFA
TGFB1
MYC
CCNA2
MT-ND6
MMP1
STAT1
HMGCR
NR3C2
GSR
HMOX1
MMP3
PPP3CB
GSTM1
IL10
MAPK1
PLAT
MDM2
EGFR
SOD1
ERBB2
IL4
AKR1B1
TRPC6
IGHG1
RUNX2
CXCL10
TP53
CASP9
CDKN1A
CAT
RASA1

IL1B
NFKBIA
IGFBP3
ALB
SLC6A3
LTA4H
PLAU
PTEN
ALOX5
RXRA
CLDN4
PON1
E2F1
APOB
CASP8
PPARG
CXCL8
SELE
THBD
MAPK14
MPO
PCNA
CHUK
STAT3
SLPI
ADRB2
CTSD
TFRC
MCL1
CCL2
IL6
CASP3
PARP1
MGAM
KDR
IGF2
PIK3CG
IL2
IFNG
FOS
HSPA5
ICAM1
BCL2L1
MAPK3
HIF1A
RELA
NOS3
SERPINE1
VCAM1
INSR
AKT1
PTGS1
F3
PYGM
CDKN2A
JUN
BAX
PRKCA
CD40LG
CYP3A4
SCN5A

APP
MMP9
TOP1
ADIPOQ

Supplementary Table 5. Detail information on the known ligands of the top five targets.

Target	Known ligand	Full name of ligand	Molecular weight	Molecular formula	Affinity energy (kcal/mol)
AKT1	UC8	~{N}-methyl-6-[4-[[4-[2-oxidanylidene-6- (propanoylamino)-3~{H}-benzimidazol-1- yl]piperidin-1-yl]methyl]phenyl]-5-phenyl- pyridine-3-carboxamide	588.7	C35H36N6O3	−6.2
TRPC6	POV	(2S)-3-(hexadecanoyloxy)-2- [(9Z)-octadec-9- enoyloxy]propyl 2-(trimethylammonio)ethyl phosphate	760.08	C42H82NO8P	−8.0
STAT3	KQV	[(2-{[(5S,8S,10aR)-3-acetyl-8-({(2S)-5-amino-1-[(diphenylmethyl)amino]- 1,5-dioxopentan-2- yl}carbamoyl)-6-oxodecahydropyrrolo[1,2-a][1,5]diazocin-5- yl]carbamoyl}-1H-indol-5- yl)(difluoro)methyl]phosph onic acid (non-preferred name)	835.79	40H44F2N7O9	−7.1
PPP3CA	PGE	TRIETHYLENE GLYCOL	150.17	C6H14O4	−7.8
NFATC	3ANP	PHOSPHOAMINOPHOSP HONIC ACID-ADENYLATE ESTER	506.2	10H17N6O12P	−8.2
