# Supplementary material for: Exploring the therapeutic mechanism of Yuebi decoction on nephrotic syndrome based on network pharmacology and experimental study
Source: Aging (Albany NY). 2024 Sep 20;16(18):12623–50. doi: 10.18632/aging.206116 (PMC11466484; doi:10.18632/aging.206116)
Supplement: Supplementary Table 4 [file aging-16-206116-s002.pdf]

Supplementary Table 4. Detailed information on GO enrichment analysis.

Category	Term	Count	%	PValue	Genes	List Total	Pop Hits
GOTERM_BP_D	GO:00459	37	31.09243697	5.04E-16	APP, ADI	119	1201
GOTERM_BP_D	GO:00106	34	28.57142857	8.81E-25	CRP, APP	119	513
GOTERM_BP_D	GO:00430	31	26.05042017	5.17E-21	CDKN1A	119	526
GOTERM_BP_D	GO:00458	30	25.21008403	2.49E-16	HIF1A, R	119	716
GOTERM_BP_D	GO:00424	27	22.68907563	4.58E-23	CDKN1A	119	293
GOTERM_BP_D	GO:00082	25	21.00840336	2.90E-14	PTEN, RI	119	549
GOTERM_BP_D	GO:00430	23	19.32773109	5.43E-17	APP, JUN	119	328
GOTERM_BP_D	GO:00069	23	19.32773109	1.59E-11	JUN, PAR	119	607
GOTERM_BP_D	GO:00075	22	18.48739496	1.52E-20	IL10, JUN	119	194
GOTERM_BP_D	GO:00069	22	18.48739496	7.07E-14	CRP, TGI	119	411
GOTERM_BP_D	GO:00001	21	17.64705882	1.62E-06	APP, JUN	119	967
GOTERM_BP_D	GO:00071	21	17.64705882	8.30E-05	IL10, CXC	119	1267
GOTERM_BP_D	GO:00303	19	15.96638655	2.22E-14	TGFB1, H	119	256
GOTERM_BP_D	GO:00094	18	15.12605042	1.17E-13	IL10, JUN	119	241
GOTERM_BP_D	GO:00015	18	15.12605042	2.43E-13	JUN, CXC	119	252
GOTERM_BP_D	GO:00019	17	14.28571429	1.83E-13	APP, TGI	119	209
GOTERM_BP_D	GO:00082	17	14.28571429	2.10E-08	IL10, APP	119	459
GOTERM_BP_D	GO:00714	16	13.44537815	7.48E-15	TRPC6, P	119	140
GOTERM_BP_D	GO:00324	16	13.44537815	3.79E-14	JUN, VCA	119	156
GOTERM_BP_D	GO:00106	16	13.44537815	6.72E-10	APP, CDI	119	308
GOTERM_BP_D	GO:00323	15	12.60504202	6.91E-15	TGFB1, P	119	112
GOTERM_BP_D	GO:00517	15	12.60504202	8.68E-09	CDKN1A	119	316
GOTERM_BP_D	GO:00064	15	12.60504202	2.39E-06	APP, TGI	119	501
GOTERM_BP_D	GO:00016	14	11.76470588	9.16E-11	TGFB1, V	119	182
GOTERM_BP_D	GO:00712	14	11.76470588	1.90E-10	IL10, CXC	119	193
GOTERM_BP_D	GO:00454	13	10.92436975	3.26E-11	VCAM1, C	119	133
GOTERM_BP_D	GO:00713	13	10.92436975	7.68E-11	VCAM1, C	119	143
GOTERM_BP_D	GO:00457	13	10.92436975	3.80E-10	CXCL8, N	119	164
GOTERM_BP_D	GO:00703	13	10.92436975	1.61E-08	APP, JUN	119	228
GOTERM_BP_D	GO:00065	13	10.92436975	8.06E-06	MMP1, M	119	409
GOTERM_BP_D	GO:00458	13	10.92436975	2.72E-04	JUN, TGI	119	590
GOTERM_BP_D	GO:00435	12	10.08403361	3.60E-09	IL10, JUN	119	158
GOTERM_BP_D	GO:00434	12	10.08403361	4.11E-09	APP, IL6,	119	160
GOTERM_BP_D	GO:00075	12	10.08403361	1.01E-07	IL1A, CD	119	218
GOTERM_BP_D	GO:00069	12	10.08403361	1.06E-06	CASP9, C	119	276
GOTERM_BP_D	GO:00071	12	10.08403361	6.30E-04	COL1A1,	119	557
GOTERM_BP_D	GO:00486	11	9.243697479	1.48E-11	IL6, TGFI	119	70
GOTERM_BP_D	GO:00096	11	9.243697479	1.36E-10	CDKN1A	119	87
GOTERM_BP_D	GO:00355	11	9.243697479	4.52E-04	CXCL8, T	119	450
GOTERM_BP_D	GO:00069	11	9.243697479	8.99E-04	IL10, IL4,	119	492
GOTERM_BP_D	GO:00712	10	8.403361345	2.66E-12	JUN, CHU	119	41

GOTERM_BP_D	GO:19028	10	8.403361345	1.23E-11	IL10, JUN	119	48
GOTERM_BP_D	GO:00425	10	8.403361345	3.16E-11	MT-ND6,	119	53
GOTERM_BP_D	GO:00514	10	8.403361345	7.08E-10	CASP9, A	119	74
GOTERM_BP_D	GO:00328	10	8.403361345	2.28E-08	PARP1, S	119	109
GOTERM_BP_D	GO:00510	10	8.403361345	6.22E-07	APP, TGF	119	160
GOTERM_BP_D	GO:00421	10	8.403361345	7.65E-07	NFKBIA,	119	164
GOTERM_BP_D	GO:00429	10	8.403361345	1.72E-05	CASP9, C	119	240
GOTERM_BP_D	GO:00071	10	8.403361345	1.10E-04	CXCL10,	119	305
GOTERM_BP_D	GO:00346	9	7.56302521	5.20E-11	JUN, CHU	119	37
GOTERM_BP_D	GO:00316	9	7.56302521	8.21E-11	NFKBIA,	119	39
GOTERM_BP_D	GO:00015	9	7.56302521	2.31E-10	MYC, MM	119	44
GOTERM_BP_D	GO:00340	9	7.56302521	7.99E-10	PPP3CB, J	119	51
GOTERM_BP_D	GO:00481	9	7.56302521	2.01E-09	CCNA2, C	119	57
GOTERM_BP_D	GO:00714	9	7.56302521	5.90E-09	PCNA, N	119	65
GOTERM_BP_D	GO:00019	9	7.56302521	2.31E-08	IL10, JUN	119	77
GOTERM_BP_D	GO:00434	9	7.56302521	3.14E-08	APP, TGF	119	80
GOTERM_BP_D	GO:00331	9	7.56302521	8.70E-08	APP, IL6,	119	91
GOTERM_BP_D	GO:00510	9	7.56302521	6.97E-07	IL10, PPP	119	119
GOTERM_BP_D	GO:00518	9	7.56302521	1.21E-06	APP, TGF	119	128
GOTERM_BP_D	GO:00192	9	7.56302521	5.34E-06	IL1A, IL6,	119	156
GOTERM_BP_D	GO:00181	9	7.56302521	1.58E-05	CHUK, B	119	181
GOTERM_BP_D	GO:00431	9	7.56302521	2.42E-05	IL1A, CAS	119	192
GOTERM_BP_D	GO:00971	8	6.722689076	1.58E-09	IL4, IL1A,	119	35
GOTERM_BP_D	GO:00350	8	6.722689076	4.28E-09	MT-ND6,	119	40
GOTERM_BP_D	GO:00974	8	6.722689076	6.13E-09	IL10, IL6,	119	42
GOTERM_BP_D	GO:00515	8	6.722689076	1.62E-08	COL1A1,	119	48
GOTERM_BP_D	GO:00435	8	6.722689076	2.18E-08	TGFB1, N	119	50
GOTERM_BP_D	GO:00327	8	6.722689076	1.41E-07	IL6, NOS2	119	65
GOTERM_BP_D	GO:00356	8	6.722689076	1.74E-07	NOS2, TF	119	67
GOTERM_BP_D	GO:00000	8	6.722689076	3.48E-07	PPP3CA,	119	74
GOTERM_BP_D	GO:00082	8	6.722689076	7.05E-07	IL4, APP,	119	82
GOTERM_BP_D	GO:00712	8	6.722689076	1.06E-06	COL1A1,	119	87
GOTERM_BP_D	GO:00507	8	6.722689076	5.13E-06	NFKBIA,	119	110
GOTERM_BP_D	GO:00425	8	6.722689076	6.50E-06	IL6, ALO	119	114
GOTERM_BP_D	GO:00016	8	6.722689076	1.19E-05	COL1A1,	119	125
GOTERM_BP_D	GO:00001	8	6.722689076	1.39E-05	TGFB1, M	119	128
GOTERM_BP_D	GO:00017	8	6.722689076	3.43E-04	NOS3, M	119	213
GOTERM_BP_D	GO:00063	8	6.722689076	6.99E-04	JUN, PAR	119	240
GOTERM_BP_D	GO:00103	7	5.882352941	1.97E-08	IL1A, CX	119	29
GOTERM_BP_D	GO:00359	7	5.882352941	5.45E-08	VCAM1, I	119	34
GOTERM_BP_D	GO:19047	7	5.882352941	3.12E-07	IL10, CD	119	45
GOTERM_BP_D	GO:00454	7	5.882352941	5.25E-07	APP, IFN	119	49
GOTERM_BP_D	GO:00086	7	5.882352941	5.94E-07	CASP9, B	119	50

GOTERM_BP_D	GO:00714	7	5.882352941	1.06E-06	TGFB1, C	119	55
GOTERM_BP_D	GO:00313	7	5.882352941	1.06E-06	TGFB1, I	119	55
GOTERM_BP_D	GO:00346	7	5.882352941	1.61E-06	CASP9, P	119	59
GOTERM_BP_D	GO:00435	7	5.882352941	2.38E-06	CASP9, A	119	63
GOTERM_BP_D	GO:00076	7	5.882352941	4.44E-06	CLDN4, J	119	70
GOTERM_BP_D	GO:00506	7	5.882352941	5.24E-06	TGFB1, M	119	72
GOTERM_BP_D	GO:00075	7	5.882352941	7.18E-06	VCAM1,	119	76
GOTERM_BP_D	GO:00069	7	5.882352941	1.12E-05	CASP9, C	119	82
GOTERM_BP_D	GO:00140	7	5.882352941	1.20E-05	EGF, INS	119	83
GOTERM_BP_D	GO:00985	7	5.882352941	1.47E-05	CXCL10,	119	86
GOTERM_BP_D	GO:00713	7	5.882352941	1.91E-05	CXCL8, M	119	90
GOTERM_BP_D	GO:00507	7	5.882352941	2.60E-05	IL6, TGF	119	95
GOTERM_BP_D	GO:00420	7	5.882352941	4.11E-05	COL1A1,	119	103
GOTERM_BP_D	GO:00327	7	5.882352941	4.11E-05	IL1A, API	119	103
GOTERM_BP_D	GO:00069	7	5.882352941	9.64E-05	APP, HM	119	120
GOTERM_BP_D	GO:00507	7	5.882352941	1.44E-04	IL10, IL4,	119	129
GOTERM_BP_D	GO:00083	7	5.882352941	3.88E-04	RASA1, F	119	155
GOTERM_BP_D	GO:00100	6	5.042016807	8.61E-08	CCND1,	119	19
GOTERM_BP_D	GO:00329	6	5.042016807	1.49E-07	CRP, APP	119	21
GOTERM_BP_D	GO:00105	6	5.042016807	1.00E-06	IL1A, IL6,	119	30
GOTERM_BP_D	GO:00466	6	5.042016807	1.40E-06	CASP9, C	119	32
GOTERM_BP_D	GO:19001	6	5.042016807	1.64E-06	TGFB1, T	119	33
GOTERM_BP_D	GO:20012	6	5.042016807	3.39E-06	IL1A, IL1	119	38
GOTERM_BP_D	GO:00069	6	5.042016807	5.63E-06	CRP, IL6,	119	42
GOTERM_BP_D	GO:00075	6	5.042016807	6.34E-06	IL1B, MM	119	43
GOTERM_BP_D	GO:00507	6	5.042016807	9.91E-06	IL6, PPP3	119	47
GOTERM_BP_D	GO:00713	6	5.042016807	9.91E-06	COL1A1,	119	47
GOTERM_BP_D	GO:00517	6	5.042016807	1.22E-05	IL1A, TG	119	49
GOTERM_BP_D	GO:00434	6	5.042016807	1.80E-05	TGFB1, I	119	53
GOTERM_BP_D	GO:00105	6	5.042016807	1.97E-05	IL10, STA	119	54
GOTERM_BP_D	GO:00148	6	5.042016807	2.36E-05	IL10, IL6,	119	56
GOTERM_BP_D	GO:00072	6	5.042016807	3.59E-05	NFKBIA,	119	61
GOTERM_BP_D	GO:00096	6	5.042016807	4.20E-05	COL1A1,	119	63
GOTERM_BP_D	GO:00327	6	5.042016807	4.89E-05	APP, IL6,	119	65
GOTERM_BP_D	GO:00421	6	5.042016807	4.89E-05	IL4, IL6,	119	65
GOTERM_BP_D	GO:00513	6	5.042016807	6.08E-05	IL10, IL6,	119	68
GOTERM_BP_D	GO:00105	6	5.042016807	6.08E-05	EGF, KD	119	68
GOTERM_BP_D	GO:00082	6	5.042016807	1.32E-04	NOS3, H	119	80
GOTERM_BP_D	GO:00457	6	5.042016807	1.57E-04	IL6, CDK	119	83
GOTERM_BP_D	GO:00015	6	5.042016807	1.76E-04	COL1A1,	119	85
GOTERM_BP_D	GO:00303	6	5.042016807	2.55E-04	ERBB2, B	119	92
GOTERM_BP_D	GO:00071	6	5.042016807	3.93E-04	JUN, TGF	119	101
GOTERM_BP_D	GO:00066	6	5.042016807	4.31E-04	NFKBIA,	119	103

GOTERM_BP_D	GO:00327	6	5.042016807	5.35E-04	IL1A, APl	119	108
GOTERM_BP_D	GO:00069	6	5.042016807	9.65E-04	CXCL10, (	119	123
GOTERM_BP_D	GO:00303	6	5.042016807	0.001113182	CDKN1A	119	127
GOTERM_BP_D	GO:00301	6	5.042016807	0.003493987	COL1A1,	119	165
GOTERM_BP_D	GO:00301	6	5.042016807	0.004377391	TRPC6, C	119	174
GOTERM_BP_D	GO:00082	6	5.042016807	0.005789142	JUN, MY(	119	186
GOTERM_BP_D	GO:00072	6	5.042016807	0.010868971	INSR, ER	119	217
GOTERM_BP_D	GO:00516	6	5.042016807	0.013935396	IL6, IFNG	119	231
GOTERM_BP_D	GO:00104	6	5.042016807	0.020458325	IL10, APP	119	255
GOTERM_BP_D	GO:00519	5	4.201680672	9.18E-07	THBD, PI	119	13
GOTERM_BP_D	GO:00511	5	4.201680672	2.30E-06	CHUK, C	119	16
GOTERM_BP_D	GO:19040	5	4.201680672	2.30E-06	CASP9, C	119	16
GOTERM_BP_D	GO:00352	5	4.201680672	3.84E-06	IL1A, IL1	119	18
GOTERM_BP_D	GO:00550	5	4.201680672	6.02E-06	COL1A1,	119	20
GOTERM_BP_D	GO:00359	5	4.201680672	6.02E-06	NFKBIA,	119	20
GOTERM_BP_D	GO:00101	5	4.201680672	6.02E-06	THBD, CI	119	20
GOTERM_BP_D	GO:19028	5	4.201680672	7.40E-06	APP, TGI	119	21
GOTERM_BP_D	GO:00018	5	4.201680672	9.00E-06	JUN, BCL	119	22
GOTERM_BP_D	GO:00602	5	4.201680672	1.08E-05	IL6, MYC	119	23
GOTERM_BP_D	GO:00458	5	4.201680672	1.30E-05	APP, IFN	119	24
GOTERM_BP_D	GO:00302	5	4.201680672	1.53E-05	CASP8, IF	119	25
GOTERM_BP_D	GO:00108	5	4.201680672	2.11E-05	NFKBIA,	119	27
GOTERM_BP_D	GO:19020	5	4.201680672	2.45E-05	NOS3, SE	119	28
GOTERM_BP_D	GO:20012	5	4.201680672	2.45E-05	BCL2, AK	119	28
GOTERM_BP_D	GO:00703	5	4.201680672	2.83E-05	IL1B, GS	119	29
GOTERM_BP_D	GO:00108	5	4.201680672	3.25E-05	APP, TGI	119	30
GOTERM_BP_D	GO:00458	5	4.201680672	3.25E-05	IL1A, EG	119	30
GOTERM_BP_D	GO:00432	5	4.201680672	3.71E-05	CHUK, C	119	31
GOTERM_BP_D	GO:00714	5	4.201680672	3.71E-05	CDKN1A	119	31
GOTERM_BP_D	GO:00071	5	4.201680672	4.22E-05	VCAM1, (	119	32
GOTERM_BP_D	GO:00346	5	4.201680672	6.06E-05	CASP8, C	119	35
GOTERM_BP_D	GO:20003	5	4.201680672	6.06E-05	CDKN1A	119	35
GOTERM_BP_D	GO:00486	5	4.201680672	6.78E-05	IFNG, N(	119	36
GOTERM_BP_D	GO:20003	5	4.201680672	6.78E-05	IL10, IL4,	119	36
GOTERM_BP_D	GO:00715	5	4.201680672	7.57E-05	CASP9, T(	119	37
GOTERM_BP_D	GO:00459	5	4.201680672	8.42E-05	AKT1, HN	119	38
GOTERM_BP_D	GO:00705	5	4.201680672	1.03E-04	APP, IL1E	119	40
GOTERM_BP_D	GO:00303	5	4.201680672	1.14E-04	TGFB1, T	119	41
GOTERM_BP_D	GO:00094	5	4.201680672	1.25E-04	CXCL10, (	119	42
GOTERM_BP_D	GO:19900	5	4.201680672	1.25E-04	APP, HSP	119	42
GOTERM_BP_D	GO:19012	5	4.201680672	1.37E-04	APP, CAS	119	43
GOTERM_BP_D	GO:00423	5	4.201680672	1.51E-04	TGFB1, II	119	44
GOTERM_BP_D	GO:00308	5	4.201680672	1.64E-04	IL4, CDK	119	45

GOTERM_BP_D	GO:00327	5	4.201680672	1.64E-04	APP, IL6,	119	45
GOTERM_BP_D	GO:00434	5	4.201680672	1.64E-04	IL1B, GST	119	45
GOTERM_BP_D	GO:00311	5	4.201680672	1.64E-04	CCNA2, C	119	45
GOTERM_BP_D	GO:00075	5	4.201680672	1.95E-04	CCND1, I	119	47
GOTERM_BP_D	GO:19046	5	4.201680672	1.95E-04	APP, VCA	119	47
GOTERM_BP_D	GO:00485	5	4.201680672	2.12E-04	BCL2, MA	119	48
GOTERM_BP_D	GO:00107	5	4.201680672	2.29E-04	COL1A1,	119	49
GOTERM_BP_D	GO:00506	5	4.201680672	2.29E-04	EGF, KD	119	49
GOTERM_BP_D	GO:00421	5	4.201680672	2.68E-04	IL4, PPP3	119	51
GOTERM_BP_D	GO:00020	5	4.201680672	3.11E-04	TGFB1, C	119	53
GOTERM_BP_D	GO:00346	5	4.201680672	3.11E-04	IL1A, CX	119	53
GOTERM_BP_D	GO:00332	5	4.201680672	3.59E-04	NFKBIA,	119	55
GOTERM_BP_D	GO:00703	5	4.201680672	3.59E-04	EGF, MY	119	55
GOTERM_BP_D	GO:19047	5	4.201680672	3.59E-04	IL10, JUN	119	55
GOTERM_BP_D	GO:00068	5	4.201680672	4.40E-04	TFRC, M	119	58
GOTERM_BP_D	GO:00713	5	4.201680672	4.40E-04	INSR, ER	119	58
GOTERM_BP_D	GO:00069	5	4.201680672	5.34E-04	IL6, IFNC	119	61
GOTERM_BP_D	GO:00076	5	4.201680672	5.68E-04	APP, PPP	119	62
GOTERM_BP_D	GO:00018	5	4.201680672	7.20E-04	IL10, IL1	119	66
GOTERM_BP_D	GO:00076	5	4.201680672	7.20E-04	APP, CAS	119	66
GOTERM_BP_D	GO:00060	5	4.201680672	7.63E-04	MYC, AD	119	67
GOTERM_BP_D	GO:00431	5	4.201680672	8.52E-04	MDM2, X	119	69
GOTERM_BP_D	GO:19012	5	4.201680672	9.00E-04	APP, IL1E	119	70
GOTERM_BP_D	GO:00436	5	4.201680672	1.00E-03	CCND1, N	119	72
GOTERM_BP_D	GO:00336	5	4.201680672	0.001052885	INSR, ER	119	73
GOTERM_BP_D	GO:00703	5	4.201680672	0.001107837	IL6, PCN	119	74
GOTERM_BP_D	GO:00971	5	4.201680672	0.001223723	CASP8, C	119	76
GOTERM_BP_D	GO:00988	5	4.201680672	0.001284734	GSTP1, A	119	77
GOTERM_BP_D	GO:00328	5	4.201680672	0.001284734	IL10, CAT	119	77
GOTERM_BP_D	GO:00019	5	4.201680672	0.001480495	TGFB1, I	119	80
GOTERM_BP_D	GO:00305	5	4.201680672	0.00162199	CXCL10,	119	82
GOTERM_BP_D	GO:00076	5	4.201680672	0.002016384	APP, PPP	119	87
GOTERM_BP_D	GO:00075	5	4.201680672	0.002102533	THBD, PI	119	88
GOTERM_BP_D	GO:00072	5	4.201680672	0.002282397	CCNA2, C	119	90
GOTERM_BP_D	GO:00316	5	4.201680672	0.002571664	CDKN2A	119	93
GOTERM_BP_D	GO:00075	5	4.201680672	0.00288517	THBD, CI	119	96
GOTERM_BP_D	GO:00713	5	4.201680672	0.003223883	NOS2, ST	119	99
GOTERM_BP_D	GO:00467	5	4.201680672	0.00346417	TFRC, IN	119	101
GOTERM_BP_D	GO:00507	5	4.201680672	0.004117583	ALOX5, X	119	106
GOTERM_BP_D	GO:00902	5	4.201680672	0.004547126	COL1A1,	119	109
GOTERM_BP_D	GO:00096	5	4.201680672	0.004849667	IFNG, CF	119	111
GOTERM_BP_D	GO:00165	5	4.201680672	0.007558199	CXCL10,	119	126
GOTERM_BP_D	GO:00163	5	4.201680672	0.008198383	STAT3, A	119	129

GOTERM_BP_D	GO:00181	5	4.201680672	0.01058629	EGF, INS	119	139
GOTERM_BP_D	GO:00467	5	4.201680672	0.025325627	INSR, ER	119	181
GOTERM_BP_D	GO:00986	5	4.201680672	0.029552228	VCAM1, I	119	190
GOTERM_BP_D	GO:00068	5	4.201680672	0.039193239	APP, OLF	119	208
GOTERM_BP_D	GO:00335	4	3.361344538	7.71E-06	IL1A, GST	119	7
GOTERM_BP_D	GO:00108	4	3.361344538	2.61E-05	NFKBIA,	119	10
GOTERM_BP_D	GO:00714	4	3.361344538	3.57E-05	MMP1, M	119	11
GOTERM_BP_D	GO:19038	4	3.361344538	4.74E-05	IL6, TGFI	119	12
GOTERM_BP_D	GO:00107	4	3.361344538	6.13E-05	NFKBIA,	119	13
GOTERM_BP_D	GO:00343	4	3.361344538	7.77E-05	ADIPOQ,	119	14
GOTERM_BP_D	GO:00302	4	3.361344538	1.19E-04	MAPK1, I	119	16
GOTERM_BP_D	GO:00903	4	3.361344538	1.19E-04	CDKN1A	119	16
GOTERM_BP_D	GO:00466	4	3.361344538	1.19E-04	IL1A, TFF	119	16
GOTERM_BP_D	GO:00481	4	3.361344538	1.43E-04	APP, IFN	119	17
GOTERM_BP_D	GO:00086	4	3.361344538	1.43E-04	CDKN2A	119	17
GOTERM_BP_D	GO:00517	4	3.361344538	1.71E-04	IFNG, ST	119	18
GOTERM_BP_D	GO:00332	4	3.361344538	1.71E-04	CXCL10,	119	18
GOTERM_BP_D	GO:00427	4	3.361344538	2.02E-04	PLAU, SE	119	19
GOTERM_BP_D	GO:00308	4	3.361344538	2.37E-04	IL10, CDI	119	20
GOTERM_BP_D	GO:00457	4	3.361344538	3.17E-04	TFRC, SP	119	22
GOTERM_BP_D	GO:00380	4	3.361344538	3.17E-04	INSR, KD	119	22
GOTERM_BP_D	GO:00020	4	3.361344538	3.63E-04	STAT1, M	119	23
GOTERM_BP_D	GO:00705	4	3.361344538	4.13E-04	PON1, CA	119	24
GOTERM_BP_D	GO:00512	4	3.361344538	4.13E-04	NFKBIA,	119	24
GOTERM_BP_D	GO:00458	4	3.361344538	4.13E-04	AKT1, PL	119	24
GOTERM_BP_D	GO:00971	4	3.361344538	4.68E-04	CASP9, C.	119	25
GOTERM_BP_D	GO:00075	4	3.361344538	4.68E-04	BCL2, TP	119	25
GOTERM_BP_D	GO:00400	4	3.361344538	4.68E-04	APP, CDI	119	25
GOTERM_BP_D	GO:00020	4	3.361344538	5.26E-04	EGF, INS	119	26
GOTERM_BP_D	GO:00466	4	3.361344538	5.89E-04	SPP1, MA	119	27
GOTERM_BP_D	GO:00350	4	3.361344538	6.57E-04	APP, TGF	119	28
GOTERM_BP_D	GO:00017	4	3.361344538	8.07E-04	IL4, APP,	119	30
GOTERM_BP_D	GO:00430	4	3.361344538	8.90E-04	BCL2, IGI	119	31
GOTERM_BP_D	GO:00518	4	3.361344538	8.90E-04	BCL2, BA	119	31
GOTERM_BP_D	GO:00423	4	3.361344538	8.90E-04	APP, EGI	119	31
GOTERM_BP_D	GO:00433	4	3.361344538	9.77E-04	NFKBIA,	119	32
GOTERM_BP_D	GO:00430	4	3.361344538	9.77E-04	PPP3CB,	119	32
GOTERM_BP_D	GO:00457	4	3.361344538	9.77E-04	JUN, PCN	119	32
GOTERM_BP_D	GO:00162	4	3.361344538	9.77E-04	IL4, KDR	119	32
GOTERM_BP_D	GO:00433	4	3.361344538	0.001070272	EGF, MY	119	33
GOTERM_BP_D	GO:00457	4	3.361344538	0.001070272	CDKN1A	119	33
GOTERM_BP_D	GO:00443	4	3.361344538	0.001070272	COL1A1,	119	33
GOTERM_BP_D	GO:00481	4	3.361344538	0.00116863	MYC, GS	119	34

GOTERM_BP_D	GO:00971	4	3.361344538	0.00116863	CASP9, C	119	34
GOTERM_BP_D	GO:00456	4	3.361344538	0.00116863	STAT1, S	119	34
GOTERM_BP_D	GO:00519	4	3.361344538	0.00116863	MYC, MA	119	34
GOTERM_BP_D	GO:00900	4	3.361344538	0.00127251	KDR, HM	119	35
GOTERM_BP_D	GO:00484	4	3.361344538	0.001382026	CTNNB1,	119	36
GOTERM_BP_D	GO:00457	4	3.361344538	0.001382026	IL6, ERBI	119	36
GOTERM_BP_D	GO:00464	4	3.361344538	0.001497291	IL10, IL6,	119	37
GOTERM_BP_D	GO:00325	4	3.361344538	0.001497291	CLDN4, T	119	37
GOTERM_BP_D	GO:00463	4	3.361344538	0.001745499	INSR, AD	119	39
GOTERM_BP_D	GO:00422	4	3.361344538	0.001745499	MT-ND6,	119	39
GOTERM_BP_D	GO:00714	4	3.361344538	0.002017977	CDKN1A	119	41
GOTERM_BP_D	GO:00305	4	3.361344538	0.002017977	MMP1, M	119	41
GOTERM_BP_D	GO:00327	4	3.361344538	0.002017977	IL4, IL6, C	119	41
GOTERM_BP_D	GO:00072	4	3.361344538	0.002017977	IFNG, ST	119	41
GOTERM_BP_D	GO:00713	4	3.361344538	0.002163568	IL10, CCN	119	42
GOTERM_BP_D	GO:00327	4	3.361344538	0.002315524	CD40LG,	119	43
GOTERM_BP_D	GO:00305	4	3.361344538	0.002315524	RXRA, TC	119	43
GOTERM_BP_D	GO:00421	4	3.361344538	0.002638904	IL10, CD4	119	45
GOTERM_BP_D	GO:00140	4	3.361344538	0.002638904	ERBB2, P	119	45
GOTERM_BP_D	GO:00421	4	3.361344538	0.002638904	NOS2, HM	119	45
GOTERM_BP_D	GO:00226	4	3.361344538	0.002810508	MMP1, M	119	46
GOTERM_BP_D	GO:00067	4	3.361344538	0.002988838	GSTM1, C	119	47
GOTERM_BP_D	GO:00071	4	3.361344538	0.003366014	TGFB1, E	119	49
GOTERM_BP_D	GO:00720	4	3.361344538	0.003984262	KDR, CT	119	52
GOTERM_BP_D	GO:00971	4	3.361344538	0.003984262	TGFB1, C	119	52
GOTERM_BP_D	GO:00094	4	3.361344538	0.004204646	NOS3, HS	119	53
GOTERM_BP_D	GO:00431	4	3.361344538	0.004204646	CASP8, S	119	53
GOTERM_BP_D	GO:00000	4	3.361344538	0.004204646	CCNA2, C	119	53
GOTERM_BP_D	GO:20000	4	3.361344538	0.004204646	CDKN1A	119	53
GOTERM_BP_D	GO:00903	4	3.361344538	0.004432301	CDKN1A	119	54
GOTERM_BP_D	GO:00192	4	3.361344538	0.004667295	MAPK1, C	119	55
GOTERM_BP_D	GO:00434	4	3.361344538	0.004909697	COL1A1,	119	56
GOTERM_BP_D	GO:00455	4	3.361344538	0.005416981	IL6, TGFI	119	58
GOTERM_BP_D	GO:20001	4	3.361344538	0.005416981	CDKN1A	119	58
GOTERM_BP_D	GO:00712	4	3.361344538	0.005681986	COL1A1,	119	59
GOTERM_BP_D	GO:00457	4	3.361344538	0.005681986	PPP3CA,	119	59
GOTERM_BP_D	GO:00713	4	3.361344538	0.005954647	APP, HSP	119	60
GOTERM_BP_D	GO:00424	4	3.361344538	0.005954647	BAX, CTN	119	60
GOTERM_BP_D	GO:00719	4	3.361344538	0.00652316	CD40LG,	119	62
GOTERM_BP_D	GO:00015	4	3.361344538	0.006819121	TGFB1, R	119	63
GOTERM_BP_D	GO:00301	4	3.361344538	0.007122955	IL6, CD40	119	64
GOTERM_BP_D	GO:00029	4	3.361344538	0.007122955	CASP9, B	119	64
GOTERM_BP_D	GO:00301	4	3.361344538	0.008417974	CASP3, M	119	68

GOTERM_BP_D	GO:00425	4	3.361344538	0.008761877	IL4, IL6, I	119	69
GOTERM_BP_D	GO:00096	4	3.361344538	0.009113922	TGFB1, M	119	70
GOTERM_BP_D	GO:00064	4	3.361344538	0.009474149	CDKN2A	119	71
GOTERM_BP_D	GO:00181	4	3.361344538	0.009474149	BCL2, MA	119	71
GOTERM_BP_D	GO:00068	4	3.361344538	0.010997602	INSR, AD	119	75
GOTERM_BP_D	GO:00320	4	3.361344538	0.010997602	CDKN1A	119	75
GOTERM_BP_D	GO:00097	4	3.361344538	0.011399268	TGFB1, C	119	76
GOTERM_BP_D	GO:00456	4	3.361344538	0.011809317	PPP3CA,	119	77
GOTERM_BP_D	GO:00327	4	3.361344538	0.011809317	IL10, IL4,	119	77
GOTERM_BP_D	GO:00301	4	3.361344538	0.015881086	IL10, IL4,	119	86
GOTERM_BP_D	GO:00076	4	3.361344538	0.016376254	APP, PTE	119	87
GOTERM_BP_D	GO:00104	4	3.361344538	0.016880032	ADIPOQ,	119	88
GOTERM_BP_D	GO:00305	4	3.361344538	0.016880032	TGFB1, P	119	88
GOTERM_BP_D	GO:00320	4	3.361344538	0.018981482	NFKBIA,	119	92
GOTERM_BP_D	GO:00712	4	3.361344538	0.018981482	JUN, HSP	119	92
GOTERM_BP_D	GO:00508	4	3.361344538	0.020084137	APP, IL6,	119	94
GOTERM_BP_D	GO:00463	4	3.361344538	0.022393539	IL1A, API	119	98
GOTERM_BP_D	GO:00063	4	3.361344538	0.029459996	CCNA2,	119	109
GOTERM_BP_D	GO:00618	4	3.361344538	0.029459996	CXCL10,	119	109
GOTERM_BP_D	GO:00701	3	2.521008403	1.11E-04	CCND1,	119	3
GOTERM_BP_D	GO:00973	3	2.521008403	2.22E-04	CXCL10,	119	4
GOTERM_BP_D	GO:00421	3	2.521008403	3.68E-04	APOB, CT	119	5
GOTERM_BP_D	GO:00022	3	2.521008403	3.68E-04	IL1A, TG	119	5
GOTERM_BP_D	GO:00380	3	2.521008403	3.68E-04	KDR, HS	119	5
GOTERM_BP_D	GO:00614	3	2.521008403	5.50E-04	HIF1A, T	119	6
GOTERM_BP_D	GO:00320	3	2.521008403	5.50E-04	CASP9, C	119	6
GOTERM_BP_D	GO:00105	3	2.521008403	7.66E-04	IL6, IL1B,	119	7
GOTERM_BP_D	GO:00107	3	2.521008403	7.66E-04	TGFB1, S	119	7
GOTERM_BP_D	GO:00604	3	2.521008403	7.66E-04	MAPK1,	119	7
GOTERM_BP_D	GO:00381	3	2.521008403	0.001017751	EGF, ER	119	8
GOTERM_BP_D	GO:00105	3	2.521008403	0.001017751	THBD, N	119	8
GOTERM_BP_D	GO:00107	3	2.521008403	0.001017751	PLAU, SE	119	8
GOTERM_BP_D	GO:00026	3	2.521008403	0.001017751	IL4, GST	119	8
GOTERM_BP_D	GO:00713	3	2.521008403	0.001017751	MDM2, B	119	8
GOTERM_BP_D	GO:00509	3	2.521008403	0.001303296	IL1A, IL1	119	9
GOTERM_BP_D	GO:00511	3	2.521008403	0.001303296	GSTM1,	119	9
GOTERM_BP_D	GO:00328	3	2.521008403	0.001622598	GSTP1, M	119	10
GOTERM_BP_D	GO:00443	3	2.521008403	0.001622598	CCNA2, S	119	10
GOTERM_BP_D	GO:19015	3	2.521008403	0.001622598	JUN, REL	119	10
GOTERM_BP_D	GO:00149	3	2.521008403	0.001622598	PLAU, BC	119	10
GOTERM_BP_D	GO:00443	3	2.521008403	0.001622598	CASP9, M	119	10
GOTERM_BP_D	GO:19037	3	2.521008403	0.001975239	PPP3CA,	119	11
GOTERM_BP_D	GO:19000	3	2.521008403	0.001975239	NOS2, A	119	11

GOTERM_BP_D	GO:19903	3	2.521008403	0.002360807	CCNA2, T	119	12
GOTERM_BP_D	GO:00509	3	2.521008403	0.002360807	KDR, F3,	119	12
GOTERM_BP_D	GO:00324	3	2.521008403	0.002360807	NFKBIA,	119	12
GOTERM_BP_D	GO:00603	3	2.521008403	0.002360807	IFNG, ST	119	12
GOTERM_BP_D	GO:20002	3	2.521008403	0.002360807	IL10, IFN	119	12
GOTERM_BP_D	GO:00194	3	2.521008403	0.002778891	NOS3, MI	119	13
GOTERM_BP_D	GO:00469	3	2.521008403	0.002778891	STAT3, B	119	13
GOTERM_BP_D	GO:00485	3	2.521008403	0.002778891	BCL2, HI	119	13
GOTERM_BP_D	GO:00107	3	2.521008403	0.002778891	APP, ADI	119	13
GOTERM_BP_D	GO:00519	3	2.521008403	0.003229085	NOS3, PT	119	14
GOTERM_BP_D	GO:19049	3	2.521008403	0.003229085	IL6, REL	119	14
GOTERM_BP_D	GO:00453	3	2.521008403	0.003710986	IL10, IL4,	119	15
GOTERM_BP_D	GO:20011	3	2.521008403	0.003710986	IL4, MYC	119	15
GOTERM_BP_D	GO:19046	3	2.521008403	0.003710986	MMP2, M	119	15
GOTERM_BP_D	GO:00331	3	2.521008403	0.003710986	PPP3CA,	119	15
GOTERM_BP_D	GO:00322	3	2.521008403	0.003710986	NFKBIA,	119	15
GOTERM_BP_D	GO:00218	3	2.521008403	0.004224196	MYC, BA	119	16
GOTERM_BP_D	GO:00193	3	2.521008403	0.004224196	ADIPOQ,	119	16
GOTERM_BP_D	GO:00431	3	2.521008403	0.004224196	TGFB1, K	119	16
GOTERM_BP_D	GO:00003	3	2.521008403	0.004224196	GSTP1, C	119	16
GOTERM_BP_D	GO:00457	3	2.521008403	0.004224196	INSR, IG	119	16
GOTERM_BP_D	GO:00442	3	2.521008403	0.00476832	MMP1, M	119	17
GOTERM_BP_D	GO:00607	3	2.521008403	0.005342966	CCND1, I	119	18
GOTERM_BP_D	GO:00510	3	2.521008403	0.005342966	APP, IFN	119	18
GOTERM_BP_D	GO:00302	3	2.521008403	0.005947745	JUN, PPA	119	19
GOTERM_BP_D	GO:00603	3	2.521008403	0.005947745	TGFB1, P	119	19
GOTERM_BP_D	GO:00507	3	2.521008403	0.005947745	TGFB1, A	119	19
GOTERM_BP_D	GO:00020	3	2.521008403	0.005947745	TGFB1, N	119	19
GOTERM_BP_D	GO:00102	3	2.521008403	0.005947745	IL1A, CD	119	19
GOTERM_BP_D	GO:20008	3	2.521008403	0.005947745	BCL2, BC	119	19
GOTERM_BP_D	GO:00514	3	2.521008403	0.006582274	CDKN1A	119	20
GOTERM_BP_D	GO:00610	3	2.521008403	0.006582274	ALOX5, S	119	20
GOTERM_BP_D	GO:00106	3	2.521008403	0.006582274	PTEN, HI	119	20
GOTERM_BP_D	GO:00071	3	2.521008403	0.006582274	EGF, AD	119	20
GOTERM_BP_D	GO:00018	3	2.521008403	0.006582274	IGF2, HII	119	20
GOTERM_BP_D	GO:19900	3	2.521008403	0.007246171	APP, CDI	119	21
GOTERM_BP_D	GO:00066	3	2.521008403	0.007246171	GSTM1, C	119	21
GOTERM_BP_D	GO:00704	3	2.521008403	0.007246171	IL1B, REI	119	21
GOTERM_BP_D	GO:00102	3	2.521008403	0.007246171	APP, CDI	119	21
GOTERM_BP_D	GO:19020	3	2.521008403	0.007246171	IFNG, CA	119	21
GOTERM_BP_D	GO:00020	3	2.521008403	0.007246171	KDR, AK	119	21
GOTERM_BP_D	GO:00068	3	2.521008403	0.00793906	NOS2, NO	119	22
GOTERM_BP_D	GO:00069	3	2.521008403	0.00793906	CDKN1A	119	22

GOTERM_BP_D	GO:00900	3	2.521008403	0.008660565	CXCL10,	119	23
GOTERM_BP_D	GO:00488	3	2.521008403	0.009410316	APOB, LI	119	24
GOTERM_BP_D	GO:00380	3	2.521008403	0.009410316	CHUK, A	119	24
GOTERM_BP_D	GO:20003	3	2.521008403	0.009410316	BCL2, HI	119	24
GOTERM_BP_D	GO:00456	3	2.521008403	0.009410316	PPP3CA,	119	24
GOTERM_BP_D	GO:00359	3	2.521008403	0.010187947	TGFB1, P	119	25
GOTERM_BP_D	GO:00723	3	2.521008403	0.010993093	E2F1, BA	119	26
GOTERM_BP_D	GO:00327	3	2.521008403	0.010993093	IL6, TGFI	119	26
GOTERM_BP_D	GO:00017	3	2.521008403	0.010993093	CASP3, B	119	26
GOTERM_BP_D	GO:00467	3	2.521008403	0.010993093	TGFB1, H	119	26
GOTERM_BP_D	GO:00026	3	2.521008403	0.011825393	IL10, IL6,	119	27
GOTERM_BP_D	GO:00488	3	2.521008403	0.012684491	NOS3, BC	119	28
GOTERM_BP_D	GO:00020	3	2.521008403	0.012684491	CTNNB1,	119	28
GOTERM_BP_D	GO:00902	3	2.521008403	0.012684491	BAX, TP5	119	28
GOTERM_BP_D	GO:00457	3	2.521008403	0.013570033	NOS2, N	119	29
GOTERM_BP_D	GO:00108	3	2.521008403	0.013570033	GSTP1, A	119	29
GOTERM_BP_D	GO:00421	3	2.521008403	0.013570033	PPP3CA,	119	29
GOTERM_BP_D	GO:00109	3	2.521008403	0.013570033	APP, CCN	119	29
GOTERM_BP_D	GO:19000	3	2.521008403	0.013570033	IL6, STAT	119	29
GOTERM_BP_D	GO:00480	3	2.521008403	0.014481668	KDR, MA	119	30
GOTERM_BP_D	GO:00359	3	2.521008403	0.015419048	MMP2, FN	119	31
GOTERM_BP_D	GO:00519	3	2.521008403	0.015419048	CCL2, PT	119	31
GOTERM_BP_D	GO:00512	3	2.521008403	0.015419048	CXCL10,	119	31
GOTERM_BP_D	GO:00487	3	2.521008403	0.015419048	PPP3CA,	119	31
GOTERM_BP_D	GO:00459	3	2.521008403	0.01638183	APP, MD	119	32
GOTERM_BP_D	GO:19007	3	2.521008403	0.01638183	IL1B, XD	119	32
GOTERM_BP_D	GO:00015	3	2.521008403	0.01638183	KDR, CT	119	32
GOTERM_BP_D	GO:00603	3	2.521008403	0.01638183	COL1A1,	119	32
GOTERM_BP_D	GO:20012	3	2.521008403	0.017369672	BCL2, BA	119	33
GOTERM_BP_D	GO:00400	3	2.521008403	0.017369672	BCL2, IG	119	33
GOTERM_BP_D	GO:00433	3	2.521008403	0.018382237	NFKBIA,	119	34
GOTERM_BP_D	GO:00322	3	2.521008403	0.018382237	MAPK1,	119	34
GOTERM_BP_D	GO:00080	3	2.521008403	0.018382237	CXCL10,	119	34
GOTERM_BP_D	GO:00508	3	2.521008403	0.018382237	ADIPOQ,	119	34
GOTERM_BP_D	GO:00459	3	2.521008403	0.019419191	IL10, TGI	119	35
GOTERM_BP_D	GO:00700	3	2.521008403	0.019419191	BCL2, BA	119	35
GOTERM_BP_D	GO:00100	3	2.521008403	0.019419191	VCAM1, I	119	35
GOTERM_BP_D	GO:00725	3	2.521008403	0.0204802	BCL2, TP	119	36
GOTERM_BP_D	GO:00074	3	2.521008403	0.0204802	CTNNB1,	119	36
GOTERM_BP_D	GO:20012	3	2.521008403	0.021564938	IL4, GSTI	119	37
GOTERM_BP_D	GO:00019	3	2.521008403	0.022673078	NOS3, MI	119	38
GOTERM_BP_D	GO:00018	3	2.521008403	0.023804299	PPARG, N	119	39
GOTERM_BP_D	GO:00019	3	2.521008403	0.023804299	STAT1, A	119	39

GOTERM_BP_D	GO:00106	3	2.521008403	0.023804299	JUN, IFN	119	39
GOTERM_BP_D	GO:00330	3	2.521008403	0.023804299	BCL2, CT	119	39
GOTERM_BP_D	GO:00086	3	2.521008403	0.023804299	CASP8, B	119	39
GOTERM_BP_D	GO:00019	3	2.521008403	0.02495828	PPP3CA,	119	40
GOTERM_BP_D	GO:00018	3	2.521008403	0.026134706	ALB, HSP	119	41
GOTERM_BP_D	GO:20006	3	2.521008403	0.026134706	KDR, CT	119	41
GOTERM_BP_D	GO:00420	3	2.521008403	0.027333263	PPP3CB,	119	42
GOTERM_BP_D	GO:00302	3	2.521008403	0.027333263	PPP3CB,	119	42
GOTERM_BP_D	GO:00072	3	2.521008403	0.02855364	BAX, AK	119	43
GOTERM_BP_D	GO:00454	3	2.521008403	0.02855364	NOS2, N	119	43
GOTERM_BP_D	GO:00016	3	2.521008403	0.029795531	MYC, BC	119	44
GOTERM_BP_D	GO:00427	3	2.521008403	0.03105863	RXRA, ST	119	45
GOTERM_BP_D	GO:00705	3	2.521008403	0.03105863	FN1, HSP	119	45
GOTERM_BP_D	GO:00018	3	2.521008403	0.032342635	TGFB1, C	119	46
GOTERM_BP_D	GO:00341	3	2.521008403	0.032342635	CDKN1A	119	46
GOTERM_BP_D	GO:00421	3	2.521008403	0.034972173	IL10, TG	119	48
GOTERM_BP_D	GO:00031	3	2.521008403	0.034972173	JUN, HIF	119	48
GOTERM_BP_D	GO:00516	3	2.521008403	0.036317116	CASP8, H	119	49
GOTERM_BP_D	GO:00457	3	2.521008403	0.036317116	CCND1,	119	49
GOTERM_BP_D	GO:00085	3	2.521008403	0.037681787	APP, HM	119	50
GOTERM_BP_D	GO:00507	3	2.521008403	0.037681787	IFNG, IL	119	50
GOTERM_BP_D	GO:00480	3	2.521008403	0.039065897	RASA1, M	119	51
GOTERM_BP_D	GO:19000	3	2.521008403	0.039065897	CCND1,	119	51
GOTERM_BP_D	GO:00325	3	2.521008403	0.040469163	RXRA, T	119	52
GOTERM_BP_D	GO:00095	3	2.521008403	0.040469163	BAX, AP	119	52
GOTERM_BP_D	GO:00071	3	2.521008403	0.040469163	VCAM1,	119	52
GOTERM_BP_D	GO:00230	2	1.680672269	0.012218685	PPP3CA,	119	2
GOTERM_BP_D	GO:00148	2	1.680672269	0.012218685	NOS3, H	119	2
GOTERM_BP_D	GO:00068	2	1.680672269	0.012218685	BCL2, BA	119	2
GOTERM_BP_D	GO:00713	2	1.680672269	0.012218685	COL1A1,	119	2
GOTERM_BP_D	GO:00970	2	1.680672269	0.012218685	BCL2, BC	119	2
GOTERM_BP_D	GO:19001	2	1.680672269	0.018272398	IL10, IL2	119	3
GOTERM_BP_D	GO:00029	2	1.680672269	0.018272398	IL10, BA	119	3
GOTERM_BP_D	GO:00613	2	1.680672269	0.018272398	MAPK1,	119	3
GOTERM_BP_D	GO:00605	2	1.680672269	0.018272398	IFNG, IL	119	3
GOTERM_BP_D	GO:00016	2	1.680672269	0.018272398	IL1A, IL1	119	3
GOTERM_BP_D	GO:00466	2	1.680672269	0.018272398	BCL2, BA	119	3
GOTERM_BP_D	GO:20006	2	1.680672269	0.018272398	IL6, STA	119	3
GOTERM_BP_D	GO:00033	2	1.680672269	0.018272398	STAT1, C	119	3
GOTERM_BP_D	GO:00190	2	1.680672269	0.018272398	MYC, IN	119	3
GOTERM_BP_D	GO:00312	2	1.680672269	0.018272398	MAPK14,	119	3
GOTERM_BP_D	GO:00148	2	1.680672269	0.018272398	IL10, CA	119	3
GOTERM_BP_D	GO:00026	2	1.680672269	0.018272398	MPO, PI	119	3

GOTERM_BP_D	GO:00344	2	1.680672269	0.018272398	IL10, SOD	119	3
GOTERM_BP_D	GO:00718	2	1.680672269	0.018272398	CASP9, C.	119	3
GOTERM_BP_D	GO:00003	2	1.680672269	0.024289323	CCND1, M	119	4
GOTERM_BP_D	GO:00343	2	1.680672269	0.024289323	CASP9, C.	119	4
GOTERM_BP_D	GO:00459	2	1.680672269	0.024289323	ADRB2, F	119	4
GOTERM_BP_D	GO:00904	2	1.680672269	0.024289323	CDKN1A	119	4
GOTERM_BP_D	GO:00451	2	1.680672269	0.024289323	IL10, IL4	119	4
GOTERM_BP_D	GO:00901	2	1.680672269	0.024289323	MAPK1, M	119	4
GOTERM_BP_D	GO:00708	2	1.680672269	0.024289323	MAPK1, M	119	4
GOTERM_BP_D	GO:00335	2	1.680672269	0.024289323	RELA, EC	119	4
GOTERM_BP_D	GO:00485	2	1.680672269	0.024289323	KDR, BA	119	4
GOTERM_BP_D	GO:00015	2	1.680672269	0.024289323	HSPA5, C	119	4
GOTERM_BP_D	GO:19906	2	1.680672269	0.024289323	MYC, FO	119	4
GOTERM_BP_D	GO:00727	2	1.680672269	0.024289323	MDM2, T	119	4
GOTERM_BP_D	GO:19016	2	1.680672269	0.024289323	GSTM1, C	119	4
GOTERM_BP_D	GO:00459	2	1.680672269	0.030269681	ADIPOQ,	119	5
GOTERM_BP_D	GO:00323	2	1.680672269	0.030269681	NOS2, PT	119	5
GOTERM_BP_D	GO:19052	2	1.680672269	0.030269681	EGF, VEC	119	5
GOTERM_BP_D	GO:00023	2	1.680672269	0.030269681	BCL2, TP	119	5
GOTERM_BP_D	GO:19025	2	1.680672269	0.030269681	IL6, BAX	119	5
GOTERM_BP_D	GO:00316	2	1.680672269	0.030269681	IL1B, PTC	119	5
GOTERM_BP_D	GO:19038	2	1.680672269	0.030269681	IL4, PPAF	119	5
GOTERM_BP_D	GO:00363	2	1.680672269	0.030269681	KDR, VE	119	5
GOTERM_BP_D	GO:00605	2	1.680672269	0.030269681	CDKN1A	119	5
GOTERM_BP_D	GO:00716	2	1.680672269	0.030269681	IL4, TGFI	119	5
GOTERM_BP_D	GO:20004	2	1.680672269	0.030269681	ADIPOQ,	119	5
GOTERM_BP_D	GO:00725	2	1.680672269	0.030269681	MAPK1, M	119	5
GOTERM_BP_D	GO:00226	2	1.680672269	0.030269681	VCAM1, I	119	5
GOTERM_BP_D	GO:00330	2	1.680672269	0.036213693	IL1A, IL1	119	6
GOTERM_BP_D	GO:00517	2	1.680672269	0.036213693	IFNG, NC	119	6
GOTERM_BP_D	GO:00330	2	1.680672269	0.036213693	CDKN2A	119	6
GOTERM_BP_D	GO:00436	2	1.680672269	0.036213693	HMOX1,	119	6
GOTERM_BP_D	GO:20004	2	1.680672269	0.036213693	ADIPOQ,	119	6
GOTERM_BP_D	GO:19047	2	1.680672269	0.036213693	IFNG, CT	119	6
GOTERM_BP_D	GO:00101	2	1.680672269	0.036213693	IL1A, CA	119	6
GOTERM_BP_D	GO:00435	2	1.680672269	0.036213693	PARP1, T	119	6
GOTERM_BP_D	GO:19048	2	1.680672269	0.036213693	IL6, TGFI	119	6
GOTERM_BP_D	GO:00313	2	1.680672269	0.042121578	IL1B, PTC	119	7
GOTERM_BP_D	GO:00306	2	1.680672269	0.042121578	MAPK1, M	119	7
GOTERM_BP_D	GO:00713	2	1.680672269	0.042121578	CCNA2, F	119	7
GOTERM_BP_D	GO:00310	2	1.680672269	0.042121578	INSR, IGI	119	7
GOTERM_BP_D	GO:19001	2	1.680672269	0.042121578	APP, MM	119	7
GOTERM_CC_D	GO:00058	59	49.57983193	1.64E-07	APP, CDF	119	5477

GOTERM_CC_I	GO:00057	57	47.89915966	2.01E-06	APP, PTE	119	5570
GOTERM_CC_I	GO:00056	57	47.89915966	1.58E-05	APP, CDI	119	5930
GOTERM_CC_I	GO:00056	51	42.85714286	2.90E-21	APP, CXC	119	1936
GOTERM_CC_I	GO:00058	51	42.85714286	1.95E-05	APP, TFR	119	5066
GOTERM_CC_I	GO:00056	48	40.33613445	1.75E-07	CDKN1A	119	3939
GOTERM_CC_I	GO:00055	47	39.49579832	2.24E-16	CRP, APP	119	2127
GOTERM_CC_I	GO:00700	35	29.41176471	6.00E-08	APP, PCN	119	2218
GOTERM_CC_I	GO:00057	27	22.68907563	1.39E-07	MT-ND6,	119	1437
GOTERM_CC_I	GO:00160	26	21.8487395	0.005041827	APP, TFR	119	2547
GOTERM_CC_I	GO:00329	23	19.32773109	8.38E-11	APP, CDI	119	703
GOTERM_CC_I	GO:00099	18	15.12605042	1.49E-07	APP, TGI	119	638
GOTERM_CC_I	GO:00058	18	15.12605042	0.005810364	APP, VCA	119	1508
GOTERM_CC_I	GO:00484	17	14.28571429	7.96E-06	APP, CDI	119	763
GOTERM_CC_I	GO:00057	17	14.28571429	8.59E-04	APP, VCA	119	1142
GOTERM_CC_I	GO:00007	16	13.44537815	0.001107718	JUN, PCN	119	1057
GOTERM_CC_I	GO:00057	15	12.60504202	0.005200137	APP, VCA	119	1122
GOTERM_CC_I	GO:00057	13	10.92436975	2.08E-07	APP, HSP	119	306
GOTERM_CC_I	GO:00098	13	10.92436975	1.30E-05	VCAM1,	119	456
GOTERM_CC_I	GO:00056	12	10.08403361	9.87E-08	JUN, RXI	119	231
GOTERM_CC_I	GO:00432	11	9.243697479	5.23E-07	APP, RXI	119	217
GOTERM_CC_I	GO:00432	11	9.243697479	0.057309101	TFRC, HS	119	982
GOTERM_CC_I	GO:00057	11	9.243697479	0.08227646	HSPA5, B	119	1052
GOTERM_CC_I	GO:00451	10	8.403361345	1.52E-05	APP, CAS	119	251
GOTERM_CC_I	GO:00057	10	8.403361345	7.49E-05	APP, VCA	119	308
GOTERM_CC_I	GO:00059	9	7.56302521	0.003197252	HSPA5, P	119	424
GOTERM_CC_I	GO:00310	8	6.722689076	1.15E-07	APP, TGI	119	67
GOTERM_CC_I	GO:00059	8	6.722689076	3.04E-07	NOS3, IN	119	77
GOTERM_CC_I	GO:00905	8	6.722689076	6.49E-06	JUN, RXI	119	121
GOTERM_CC_I	GO:19048	8	6.722689076	7.62E-06	ALOX5, C	119	124
GOTERM_CC_I	GO:00100	8	6.722689076	6.67E-04	TFRC, IN	119	253
GOTERM_CC_I	GO:00057	8	6.722689076	0.002109486	IL1B, INS	119	309
GOTERM_CC_I	GO:00725	7	5.882352941	1.96E-04	IGHG1, T	119	145
GOTERM_CC_I	GO:00310	7	5.882352941	0.003920253	COL1A1,	119	259
GOTERM_CC_I	GO:00163	7	5.882352941	0.021336638	CLDN4, N	119	374
GOTERM_CC_I	GO:00306	6	5.042016807	5.24E-05	TFRC, EC	119	70
GOTERM_CC_I	GO:00301	6	5.042016807	2.71E-04	COL1A1,	119	99
GOTERM_CC_I	GO:00057	6	5.042016807	0.005018062	CASP8, B	119	191
GOTERM_CC_I	GO:00056	6	5.042016807	0.005356052	PARP1, A	119	194
GOTERM_CC_I	GO:00319	6	5.042016807	0.012952643	CCND1, C	119	241
GOTERM_CC_I	GO:00166	6	5.042016807	0.04662411	CDKN1A	119	339
GOTERM_CC_I	GO:00304	6	5.042016807	0.058033184	APP, TGI	119	361
GOTERM_CC_I	GO:00989	6	5.042016807	0.064876307	PPP3CA,	119	373
GOTERM_CC_I	GO:00430	6	5.042016807	0.068461456	CASP8, P	119	379

GOTERM_CC_I	GO:00003	5	4.201680672	8.17E-05	CCNA2, C	119	40
GOTERM_CC_I	GO:00451	5	4.201680672	0.001077046	APP, VCA	119	78
GOTERM_CC_I	GO:00300	5	4.201680672	0.007580613	PPP3CA, I	119	134
GOTERM_CC_I	GO:00163	5	4.201680672	0.049048034	TFRC, ER	119	238
GOTERM_CC_I	GO:00329	4	3.361344538	0.002365569	PARP1, C	119	46
GOTERM_CC_I	GO:00059	4	3.361344538	0.005268461	APP, TFR	119	61
GOTERM_CC_I	GO:00170	4	3.361344538	0.005764999	JUN, CCN	119	63
GOTERM_CC_I	GO:00986	4	3.361344538	0.013930406	PPP3CA, S	119	87
GOTERM_CC_I	GO:00057	4	3.361344538	0.027024272	NOS2, CA	119	112
GOTERM_CC_I	GO:00347	4	3.361344538	0.028917309	ALOX5, C	119	115
GOTERM_CC_I	GO:00059	4	3.361344538	0.039406821	CLDN4, C	119	130
GOTERM_CC_I	GO:00057	4	3.361344538	0.054192246	INSR, MA	119	148
GOTERM_CC_I	GO:00059	4	3.361344538	0.099548754	APP, CLD	119	192
GOTERM_CC_I	GO:00971	3	2.521008403	3.26E-04	PLAU, SE	119	5
GOTERM_CC_I	GO:00971	3	2.521008403	9.02E-04	BAX, BCI	119	8
GOTERM_CC_I	GO:00163	3	2.521008403	0.007700538	THBD, CI	119	23
GOTERM_CC_I	GO:00343	3	2.521008403	0.010524413	APP, PON	119	27
GOTERM_CC_I	GO:00057	3	2.521008403	0.010524413	APP, HSP	119	27
GOTERM_CC_I	GO:00099	3	2.521008403	0.034961642	CLDN4, I	119	51
GOTERM_CC_I	GO:00002	3	2.521008403	0.038806143	JUN, E2F	119	54
GOTERM_CC_I	GO:19047	3	2.521008403	0.040121935	LTA4H, C	119	55
GOTERM_CC_I	GO:00098	3	2.521008403	0.048360403	PPP3CA, (	119	61
GOTERM_CC_I	GO:00007	3	2.521008403	0.051232036	JUN, CTN	119	63
GOTERM_CC_I	GO:19041	3	2.521008403	0.052690378	HSPB1, H	119	64
GOTERM_CC_I	GO:00163	3	2.521008403	0.066452805	CLDN4, C	119	73
GOTERM_CC_I	GO:00708	3	2.521008403	0.066452805	MGAM, F	119	73
GOTERM_CC_I	GO:00055	3	2.521008403	0.098762454	COL1A1,	119	92
GOTERM_CC_I	GO:00971	2	1.680672269	0.011494997	CCND1, C	119	2
GOTERM_CC_I	GO:00705	2	1.680672269	0.011494997	CDKN1A	119	2
GOTERM_CC_I	GO:00359	2	1.680672269	0.028492279	JUN, FOS	119	5
GOTERM_CC_I	GO:00974	2	1.680672269	0.034093389	KDR, LD	119	6
GOTERM_CC_I	GO:00059	2	1.680672269	0.034093389	PPP3CA, I	119	6
GOTERM_CC_I	GO:00343	2	1.680672269	0.034093389	APP, APC	119	6
GOTERM_CC_I	GO:00082	2	1.680672269	0.039662478	PPP3CA, I	119	7
GOTERM_CC_I	GO:00312	2	1.680672269	0.039662478	CASP8, C.	119	7
GOTERM_CC_I	GO:00166	2	1.680672269	0.05070532	CTNNB1,	119	9
GOTERM_CC_I	GO:00360	2	1.680672269	0.05070532	PPP3CA, '	119	9
GOTERM_CC_I	GO:00056	2	1.680672269	0.056179433	APP, ALC	119	10
GOTERM_CC_I	GO:00432	2	1.680672269	0.061622245	PTEN, AI	119	11
GOTERM_CC_I	GO:19053	2	1.680672269	0.061622245	THBD, PI	119	11
GOTERM_CC_I	GO:00469	2	1.680672269	0.072414677	BCL2, BA	119	13
GOTERM_CC_I	GO:00343	2	1.680672269	0.077764649	APOB, LI	119	14
GOTERM_CC_I	GO:00319	2	1.680672269	0.098860299	APP, APC	119	18

GOTERM_CC_I	GO:00311	2	1.680672269	0.098860299	MAPK1,	119	18
GOTERM_CC_I	GO:00442	2	1.680672269	0.098860299	ERBB2, E	119	18
GOTERM_MF_I	GO:00055	112	94.11764706	6.80E-13	APP, TFR	119	12539
GOTERM_MF_I	GO:00428	46	38.65546218	8.24E-18	CRP, APP	119	1712
GOTERM_MF_I	GO:00198	31	26.05042017	1.78E-24	APP, PCN	119	390
GOTERM_MF_I	GO:00428	23	19.32773109	5.33E-10	APP, GST	119	713
GOTERM_MF_I	GO:00199	20	16.80672269	4.19E-10	CDKN1A	119	510
GOTERM_MF_I	GO:00036	19	15.96638655	0.001076012	APP, JUN	119	1286
GOTERM_MF_I	GO:00448	17	14.28571429	6.81E-09	CDKN1A	119	414
GOTERM_MF_I	GO:00009	16	13.44537815	0.008498451	APP, JUN	119	1207
GOTERM_MF_I	GO:00081	14	11.76470588	6.76E-10	JUN, PAR	119	209
GOTERM_MF_I	GO:00037	14	11.76470588	4.50E-05	JUN, STA	119	554
GOTERM_MF_I	GO:00082	14	11.76470588	0.003277052	PARP1, M	119	873
GOTERM_MF_I	GO:00009	14	11.76470588	0.057586113	JUN, STA	119	1279
GOTERM_MF_I	GO:00051	12	10.08403361	3.48E-08	IL10, IL4,	119	192
GOTERM_MF_I	GO:00316	12	10.08403361	3.49E-06	NFKBIA,	119	305
GOTERM_MF_I	GO:00036	12	10.08403361	1.73E-04	APP, JUN	119	467
GOTERM_MF_I	GO:00051	11	9.243697479	7.03E-08	APP, VC	119	161
GOTERM_MF_I	GO:00051	11	9.243697479	1.97E-04	CXCL10,	119	396
GOTERM_MF_I	GO:00046	10	8.403361345	6.46E-04	CCND1,	119	379
GOTERM_MF_I	GO:00020	9	7.56302521	4.23E-07	COL1A1,	119	109
GOTERM_MF_I	GO:00082	9	7.56302521	4.86E-07	CASP9, A	119	111
GOTERM_MF_I	GO:00080	9	7.56302521	9.97E-06	IL10, IL4,	119	166
GOTERM_MF_I	GO:00616	9	7.56302521	2.46E-05	JUN, PAR	119	188
GOTERM_MF_I	GO:00163	9	7.56302521	9.03E-05	CDKN1A	119	226
GOTERM_MF_I	GO:00199	9	7.56302521	2.55E-04	CCNA2,	119	263
GOTERM_MF_I	GO:00469	9	7.56302521	0.002363676	APP, CH	119	371
GOTERM_MF_I	GO:00046	9	7.56302521	0.003308896	CHUK, C	119	392
GOTERM_MF_I	GO:00012	9	7.56302521	0.009929577	JUN, MY	119	473
GOTERM_MF_I	GO:00510	8	6.722689076	5.96E-06	APP, TFR	119	110
GOTERM_MF_I	GO:00200	8	6.722689076	4.49E-05	NOS2, N	119	150
GOTERM_MF_I	GO:00469	8	6.722689076	1.10E-04	IL10, PPP	119	173
GOTERM_MF_I	GO:00082	8	6.722689076	1.23E-04	CXCL10,	119	176
GOTERM_MF_I	GO:00042	8	6.722689076	2.24E-04	PLAU, M	119	194
GOTERM_MF_I	GO:00199	7	5.882352941	2.62E-05	ERBB2, S	119	93
GOTERM_MF_I	GO:00036	7	5.882352941	9.52E-05	RXRA, ST	119	117
GOTERM_MF_I	GO:00055	7	5.882352941	0.001865979	PPP3CA,	119	205
GOTERM_MF_I	GO:00009	7	5.882352941	0.00390983	JUN, RX	119	238
GOTERM_MF_I	GO:00435	7	5.882352941	0.017374535	RXRA, M	119	328
GOTERM_MF_I	GO:00517	6	5.042016807	2.49E-06	STAT1, B	119	35
GOTERM_MF_I	GO:00474	6	5.042016807	8.22E-04	TGFB1, P	119	116
GOTERM_MF_I	GO:00048	6	5.042016807	0.006810085	THBD, C	119	189
GOTERM_MF_I	GO:00012	6	5.042016807	0.057559897	JUN, MY	119	331

GOTERM_MF_I GO:00977	5	4.201680672	8.28E-05	CDKN2A	119	37
GOTERM_MF_I GO:00012	5	4.201680672	9.21E-05	RXRA, MI	119	38
GOTERM_MF_I GO:00506	5	4.201680672	1.37E-04	NOS2, NC	119	42
GOTERM_MF_I GO:00971	5	4.201680672	9.29E-04	CASP8, CI	119	69
GOTERM_MF_I GO:00428	5	4.201680672	0.009606756	CCND1, I	119	132
GOTERM_MF_I GO:00080	5	4.201680672	0.042094575	PCNA, EI	119	208
GOTERM_MF_I GO:00514	4	3.361344538	4.75E-06	BCL2, BA	119	6
GOTERM_MF_I GO:00704	4	3.361344538	4.42E-04	JUN, PAR	119	24
GOTERM_MF_I GO:00303	4	3.361344538	0.002818169	PCNA, PA	119	45
GOTERM_MF_I GO:00169	4	3.361344538	0.003191212	RXRA, ST	119	47
GOTERM_MF_I GO:00048	4	3.361344538	0.004728976	RXRA, ST	119	54
GOTERM_MF_I GO:00055	4	3.361344538	0.005777176	IL1A, ALI	119	58
GOTERM_MF_I GO:00506	4	3.361344538	0.008968481	NOS2, NC	119	68
GOTERM_MF_I GO:00020	4	3.361344538	0.010090621	CDKN2A	119	71
GOTERM_MF_I GO:00082	4	3.361344538	0.010090621	MMP2, M	119	71
GOTERM_MF_I GO:00016	4	3.361344538	0.012134799	TFRC, LD	119	76
GOTERM_MF_I GO:00041	4	3.361344538	0.01741342	MMP1, M	119	87
GOTERM_MF_I GO:00041	4	3.361344538	0.023160624	CASP9, C.	119	97
GOTERM_MF_I GO:00047	4	3.361344538	0.034244139	INSR, ER	119	113
GOTERM_MF_I GO:00047	4	3.361344538	0.036578777	INSR, ER	119	116
GOTERM_MF_I GO:00042	4	3.361344538	0.039815875	MMP1, M	119	120
GOTERM_MF_I GO:00972	3	2.521008403	0.00136478	CASP9, C.	119	9
GOTERM_MF_I GO:00971	3	2.521008403	0.001698984	CASP9, C.	119	10
GOTERM_MF_I GO:00971	3	2.521008403	0.001698984	CASP9, C.	119	10
GOTERM_MF_I GO:00048	3	2.521008403	0.002471478	CDKN1A	119	12
GOTERM_MF_I GO:00167	3	2.521008403	0.002908886	ALOX5, I	119	13
GOTERM_MF_I GO:00012	3	2.521008403	0.004420552	E2F1, HII	119	16
GOTERM_MF_I GO:00047	3	2.521008403	0.004420552	MAPK1, I	119	16
GOTERM_MF_I GO:00047	3	2.521008403	0.005590273	MAPK1, I	119	18
GOTERM_MF_I GO:00009	3	2.521008403	0.006885652	STAT1, F(	119	20
GOTERM_MF_I GO:00350	3	2.521008403	0.010652525	PCNA, ST	119	25
GOTERM_MF_I GO:00507	3	2.521008403	0.010652525	CRP, APP	119	25
GOTERM_MF_I GO:00051	3	2.521008403	0.017117704	CASP8, CI	119	32
GOTERM_MF_I GO:00019	3	2.521008403	0.017117704	MMP2, IC	119	32
GOTERM_MF_I GO:00510	3	2.521008403	0.018148239	NFKBIA,	119	33
GOTERM_MF_I GO:00165	3	2.521008403	0.019204413	CCNA2, C	119	34
GOTERM_MF_I GO:00420	3	2.521008403	0.021392257	CXCL10, .	119	36
GOTERM_MF_I GO:00046	3	2.521008403	0.021392257	PTGS2, M	119	36
GOTERM_MF_I GO:00012	3	2.521008403	0.031099551	STAT1, H	119	44
GOTERM_MF_I GO:00435	3	2.521008403	0.032414964	TGFB1, C	119	45
GOTERM_MF_I GO:00017	3	2.521008403	0.035110213	RASA1, M	119	47
GOTERM_MF_I GO:00708	3	2.521008403	0.037889256	MYC, PPA	119	49
GOTERM_MF_I GO:00080	3	2.521008403	0.039309424	CXCL10, (	119	50

GOTERM_MF_I	GO:00480	3	2.521008403	0.049794512	EGF, IGF	119	57
GOTERM_MF_I	GO:00047	3	2.521008403	0.054563431	PPP3CA,	119	60
GOTERM_MF_I	GO:00422	3	2.521008403	0.068014499	RXRA, PF	119	68
GOTERM_MF_I	GO:00170	3	2.521008403	0.08242717	PPP3CA,	119	76
GOTERM_MF_I	GO:00314	3	2.521008403	0.095728492	STAT3, RI	119	83
GOTERM_MF_I	GO:00046	2	1.680672269	0.012506833	PTGS2, P'	119	2
GOTERM_MF_I	GO:00514	2	1.680672269	0.018701962	APP, INSI	119	3
GOTERM_MF_I	GO:00045	2	1.680672269	0.018701962	NOS2, NC	119	3
GOTERM_MF_I	GO:00506	2	1.680672269	0.024858554	RXRA, PF	119	4
GOTERM_MF_I	GO:00346	2	1.680672269	0.024858554	NOS2, NC	119	4
GOTERM_MF_I	GO:00331	2	1.680672269	0.030976844	PPP3CA,	119	5
GOTERM_MF_I	GO:00303	2	1.680672269	0.043099467	PPP3CB, S	119	7
GOTERM_MF_I	GO:00506	2	1.680672269	0.043099467	RXRA, PF	119	7
GOTERM_MF_I	GO:00317	2	1.680672269	0.049104267	STAT1, ST	119	8
GOTERM_MF_I	GO:00319	2	1.680672269	0.049104267	INSR, IGI	119	8
GOTERM_MF_I	GO:00302	2	1.680672269	0.055071703	AKT1, EC	119	9
GOTERM_MF_I	GO:00346	2	1.680672269	0.055071703	NOS2, NC	119	9
GOTERM_MF_I	GO:00165	2	1.680672269	0.066895402	APP, FN1	119	11
GOTERM_MF_I	GO:00083	2	1.680672269	0.072752123	CDK4, M.	119	12
GOTERM_MF_I	GO:00973	2	1.680672269	0.072752123	CDKN2A	119	12
GOTERM_MF_I	GO:00319	2	1.680672269	0.078572392	INSR, IGI	119	13
GOTERM_MF_I	GO:00428	2	1.680672269	0.078572392	TRPC6, R	119	13
GOTERM_MF_I	GO:00302	2	1.680672269	0.090104478	EGF, EGI	119	15
GOTERM_MF_I	GO:00051	2	1.680672269	0.090104478	IL1A, IL1I	119	15
GOTERM_MF_I	GO:00452	2	1.680672269	0.090104478	CXCL10, (	119	15
GOTERM_MF_I	GO:00051	2	1.680672269	0.090104478	PTEN, VI	119	15
GOTERM_MF_I	GO:00050	2	1.680672269	0.090104478	OLR1, LD	119	15
GOTERM_MF_I	GO:00167	2	1.680672269	0.09581674	NOS2, NC	119	16
GOTERM_MF_I	GO:00051	2	1.680672269	0.09581674	CASP8, C.	119	16
GOTERM_MF_I	GO:00051	2	1.680672269	0.09581674	INSR, IGI	119	16
